# Large-scale analyses of angiosperm *Flowering Locus T* genes reveal duplication and functional divergence in monocots

**DOI:** 10.3389/fpls.2022.1039500

**Published:** 2023-01-04

**Authors:** Hongling Liu, Xing Liu, Xiaojun Chang, Fei Chen, Zhenguo Lin, Liangsheng Zhang

**Affiliations:** ^1^ Hainan Institute of Zhejiang University, Sanya, China; ^2^ Genomics and Genetic Engineering Laboratory of Ornamental Plants, College of Agriculture and Biotechnology, Zhejiang University, Hangzhou, China; ^3^ College of Life Science, Fujian Agriculture and Forestry University, Fuzhou, China; ^4^ Laboratory of Medicinal Plant Biotechnology, School of Pharmaceutical Sciences, Zhejiang Chinese Medical University, Hangzhou, China; ^5^ Hainan Key Laboratory for Sustainable Utilization of Tropical Bioresources, College of Tropical Crops, Hainan University, Haikou, China; ^6^ Department of Biology, Saint Louis University, St Louis, MO, United States

**Keywords:** *Flowering locus T* (*FT*), monocots, gene duplication, positive selection, functional divergence

## Abstract

*FLOWERING LOCUS T* (*FT*) are well-known key genes for initiating flowering in plants. Delineating the evolutionary history and functional diversity of *FT* genes is important for understanding the diversification of flowering time and how plants adapt to the changing surroundings. We performed a comprehensive phylogenetic analysis of *FT* genes in 47 sequenced flowering plants and the 1,000 Plant Transcriptomes (1KP) database with a focus on monocots, especially cereals. We revealed the evolutionary history of *FT* genes. The *FT* genes in monocots can be divided into three clades (I, II, and III), whereas only one monophyletic group was detected in early angiosperms, magnoliids, and eudicots. Multiple rounds of whole-genome duplications (WGD) events followed by gene retention contributed to the expansion and variation of *FT* genes in monocots. Amino acid sites in the clade II and III genes were preferentially under high positive selection, and some sites located in vital domain regions are known to change functions when mutated. Clade II and clade III genes exhibited high variability in important regions and functional divergence compared with clade I genes; thus, clade I is more conserved than clade II and III. Genes in clade I displayed higher expression levels in studied organs and tissues than the clade II and III genes. The co-expression modules showed that some of the *FT* genes might have experienced neofunctionalization and subfunctionalization, such as the acquisition of environmental resistance. Overall, *FT* genes in monocots might form three clades by the ancient gene duplication, and each clade was subsequently subjected to different selection pressures and amino acid substitutions, which eventually led to different expression patterns and functional diversification. Our study provides a global picture of *FT* genes’ evolution in monocots, paving a road for investigating *FT* genes’ function in future.

## Introduction

Flowering is the most important step from vegetative to reproductive growth in flowering plants (i.e., angiosperms), which is also a major innovation of plant evolution. In the early 1930s, florigen was postulated as a key factor to induce flowering without knowing its genetic identity (Chailakhyan, 1936). In the last two decades, numerous studies have demonstrated that the FLOWERING LOCUS T (*FT*) proteins are the main component of florigen and are crucial in regulating flowering times (Wang et al., 2015; Samad et al., 2017; Sun et al., 2017). The *FT* proteins initiate early flowering *via* interacting with other transcription factors. In *Arabidopsis thaliana*, under a long-day condition, the expression of *FT* is tightly upregulated by an upstream transcription factor CONSTANS (CO) and consequently accelerates flowering. While under a short-day condition, *FT* is weakly expressed and flowering is thus deferred (Kobayashi et al., 1999; Tiwari et al., 2010). In the photoperiod pathway, light signals are received by photoreceptors and coincide with circadian rhythms to regulate the expression of the *CO* gene (Valverde et al., 2004). *FT* protein is transported from leaf to shoot apical meristem (SAM), where it interacts with intercellular receptors 14-3-3 proteins, to form a complex (Taoka et al., 2011). The complex can bind FLOWERING LOCUS D (FD) protein and activate *APETALA1* (*AP1*) and *SUPPRESSOR OF OVEREXPRESSION OF CONSTANS1* (*SOC1*) in order to induce flowering.

The *FT* genes belong to the phosphatidylethanolamine binding protein (PEBP) family, which consists of three subfamilies, *FT*, *TERMINAL FLOWER1* (*TFL1*), and *MOTHER OF FT AND TFL1* (*MFT*), all being involved in regulation of flowering time (Wang et al., 2015; Ito-Inaba et al., 2016; Mao et al., 2016). There are six PEBP members in Arabidopsis, namely, *FT*, *TFL1*, *MFT*, *TWIN SISTER OF FT* (*TSF*), *BROTHER OF FT AND TFL1* (*BFT*), and *CENTRORADIALIS homolog* (*ATC*) (Carmona et al., 2007; Hedman et al., 2009). *MFT*, which has weak activity in flowering induction, shares high sequence similarity with both *FT* and *TFL1*(Yoo et al., 2004), but it plays an important role in regulating seed germination (Xi et al., 2010). *TSF*, the closest *FT* homolog and functional redundant with *FT*, acts as a floral pathway integrator (Yamaguchi et al., 2005). *BFT* and *ATC* act as *TFL1-like* genes to inhibit floral initiation (Yoo et al., 2010; Huang et al., 2012). As for the *FT* subfamily, only one copy is present in the early angiosperm *Amborella trichopoda* (Wickland and Hanzawa, 2015; Drabešová et al., 2016), 2 in *Arabidopsis thaliana*, 13 in rice (Kojima et al., 2002; Komiya et al., 2008), 14 in maize (Danilevskaya et al., 2008), and 13 in sorghum (Wolabu et al., 2016). It is notable that the number of *FT* genes varies dramatically among flowering plants, particularly between monocots, magnoliids, and eudicots. However, the reason why Poaceae has more *FT* genes than other angiosperms is still unclear. More generally, the evolutionary history of the *FT* genes is unclear in monocots.

In this study, we focused on the evolutionary history and functional diversity of *FT* genes in monocots. Comparing early angiosperms, monocots, magnoliids, and eudicots, we found that the monocot *FT* genes were formed into three clades (I, II, and III), whereas only one clade was formed in magnoliids and eudicots. We also found that monocot clade II and III genes were subject to positive selection and some of them showed tissue-specific expression patterns, so they might be functionally different from the more conserved clade I. In addition, only *FT* genes in monocots were expressed highly under vernalization treatments but not eudicots. Our study paves a road for in-depth studies of *FT* gene functions in future and provides a good example of how gene duplication contributes to functional innovation.

## Materials and methods

### Identification of *FT* homolog genes in plant

To identify *FT* genes and their homologs in plants, we first downloaded all whole-genome sequenced species from the Phytozome database (https://phytozome.jgi.doe.gov/pz/portal.html); the data links are listed in **Supplemental Table 1**. Using the Arabidopsis *FT* gene (*AT1G65480*) as a query, BLASTp v2.10.0 (Camacho et al., 2009) was run against all protein sequences in each genome and the 1,000 Plant Transcriptome (1KP) database (Carpenter et al., 2019), with an E-value cutoff 10^-5^. Meanwhile, the PEBP domain (Pfam ID: PF01161) was searched in each genome using hmmer v3.0 (Mistry et al., 2013) with default parameters. All hits from BLAST and HMM searches were then combined and cleaned for eliminating redundancy.

### Multiple-sequence alignment and phylogenetic tree construction

**Figure 1:**
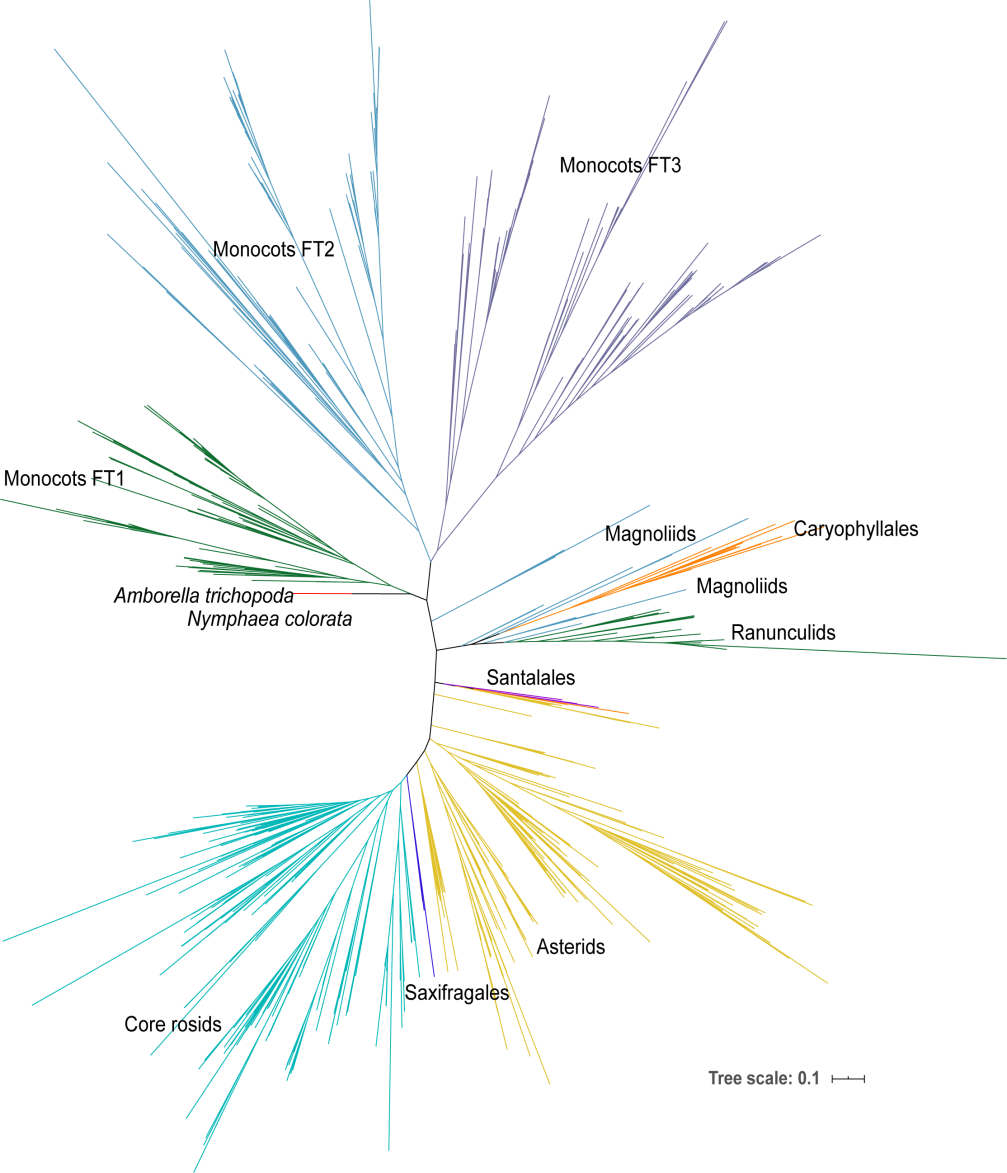
The phylogenetic tree of all *FT* genes in angiosperms. The phylogenetic tree was constructed using the ML method. The *FT* genes are from the 47-species-genome database (2 early angiosperms, 17 monocots, and 28 eudicots) and the 1KP database including early angiosperm, magnoliids, Caryophyllales, ranunculids, Santalales, Saxifragales, asterids, and core rosids.

All amino acid sequences of the PEBP domain identified from BLAST and HMM were aligned using MAFFT v7.487 (Katoh et al., 2002) (http://www.ebi.ac.uk/Tools/msa/mafft/). Then, we manually examined protein alignments in MEGA 7.0 (Kumar et al., 2016) and removed gaps. Considering that the *FT* gene is only one group of the PEBP gene family and the candidate genes identified above may contain *TFL1* and *MFT* genes, we adopted a step-by-step strategy for constructing a phylogenetic tree of *FT* genes in monocots. Firstly, we used all PEBP protein sequences to construct a PEBP phylogenetic tree using FastTree v2.0.0 (Price et al., 2009) software with default parameters. PEBP sequences were divided into three groups, and three *Selaginella moellendorffii* genes, *Smo.235975*, *scaffold00114.23*, and *Smo.235975*, were set as the outgroup separately in each gene tree (**Supplementary Figure 1**), coinciding with the three gene families, *MFT*, *TFL1*, and *FT*. Secondly, we used all sequences from early angiosperms, monocots, magnoliids, and eudicots in the *FT* family to construct another phylogenetic tree using FastTree (**Figure 1**). Knowing that the *FT* gene first emerged in angiosperms, a *Nymphaea colorata FT*-like gene was used as the outgroup. Finally, all monocots’ *FT* protein sequences were aligned together using MAFFT v7.487 and a *Picea abies FT*-like gene (*JN039333*) was set as the outgroup to construct a Bayes phylogenetic tree using the CIPRES web (https://www.phylo.org/portal2). We used MrBayes (Huelsenbeck and Ronquist, 2001; Huelsenbeck and Ronquist, 2001), setting 8,000,000 generations and four Markov chains, and the first 25% of the tree from all runs was discarded.

### Positive selection analysis

Analysis of the ratio (ω) of non-synonymous substitution (*dN*) and synonymous substitution (*dS*) is an efficient method to evaluate the type of selective pressure acting on protein-coding genes. In this study, we used PAML (Yang, 2007) to evaluate the positive selection that occurred in monocots. ω <1, = 1, and >1 indicate purifying selection, neutral evolution, and positive selection, respectively. To explore how positive selection has occurred on each clade in monocot *FT* genes, branch model was used to evaluate the ω value for the I, II, and III clades. The likelihood ratio test (LRT) was performed to compare the null model (model 0, only one ω value in the tree) and the alternative model (model 2, different ω values for foreground and background branch). The significance of the LRT was calculated by assuming that twice the difference in the log of maximum likelihoods was χ^2^ distributed, with the degrees of freedom (df) given by the difference in the number of parameters in the models (Bielawski and Yang, 2005). Positive selection was also detected using the branch site model. The test is based on the comparison between two models: a model (MA) that allows positive selection on one or more branches and a model (MA1) that does not allow positive selection on any branch. To test which amino acid residues might be under positive selection in different clades in monocots, two tests of branch site model were done; clade II was set as the foreground branch with clades I and II being the background, and clade III was set as the foreground with clades I and III being the background. The likelihood ratio test was used to compare the models, but in this case, the P-value obtained for the χ^2^ distribution of 2LRT was divided by 2 df. When the LRT suggested positive selection, the Bayes empirical Bayes (BEB) analysis was used to estimate posterior probabilities that each codon belongs to the site class of positive selection on the foreground branch. Furthermore, to check whether subclades generated in the same WGD events were under different selection pressures, we applied the branch model and branch site model for each subclade too.

### Homology modeling and evolutionary conservation analysis across residues

The structures of all *FT* proteins were solved *de novo* using the I-TASSER server (https://zhanggroup.org/I-TASSER/) (Yang et al., 2015) and along with the rice *Hd3a* structure as a templates for the modeler. Top-scoring models were chosen, and all structures in the figures were visualized by PyMOL v1.1 (http://pymol.org/). The evolutionary conservation scores across amino acid residues were calculated on the ConSurf server (https://consurf.tau.ac.il/consurf_index.php) (Ashkenazy et al., 2016) using sequence alignment including three clades of *FT* members from monocot species. The At*FT* homolog model (PDB ID: 1WKP) was used an input structure of the *FT* model, on which the variation and conservation were mapped, and all images were modified and represented using PyMOL software.

### Functional divergence calculation

The analysis of functional divergence between different *FT* paralogous clades was conducted using DIVERGE 3.0 software (Gu et al., 2013). We used type I and type II models to calculate the functional divergence coefficient θ and SE value, based on the occurrence of altered selective constraints. The test also estimates the posterior probabilities of residues that might be responsible for functional divergence. A value of 0.6 was chosen as a cutoff to measure the degree of functional divergence at the amino acid residue level.

### Gene expression analysis

Rice and maize *FT* genes’ expression data in different tissues and FT co-expression network information were downloaded from the Plant Public RNA-seq Database (PPRD, http://ipf.sustech.edu.cn/pub/plantrna/) (Yu et al., 2022). The number of libraries in different tissues has reached an order of magnitude difference, so for better presentation we have drawn the tissues with a large sample size in the main figures and drawn the tissues with a small sample size in the supplementary figures. To make the comparison more obvious, we discarded libraries as long as FPKM were all lower than 5 for genes in the heatmap. The co-expression module was collected from Yu Zhang study (2022).

Vernalization and non-vernalization RNA-seq data were downloaded from NCBI; *Aquilegia coerulea* was vernalized at 6°C (Sharma et al., 2019), *Allium sativum* vernalized at 4°C (Ben Michael et al., 2020), *Arabidopsis thaliana* vernalized at 4°C (Xi et al., 2010), *Brachypodium distachyon* vernalized at 5°C (Ream et al., 2014), *Brassica rapa* vernalized at 5°C (Jung et al., 2018), *Camelina sativa* (https://www.ncbi.nlm.nih.gov/sra/?term=SRP062374), *Brassica napus* vernalized at 5°C (Jones et al., 2018), *Dactylis glomerata* vernalized in natural environmental conditions (Feng et al., 2018), Hordeum vulgare (Deng et al., 2015), *Mimulus guttatus* vernalized at 4°C (Preston et al., 2016), and *Triticum aestivum* (https://genome.jgi.doe.gov/portal/?core=genome&query=1148146). Reads were mapped to the reference genome using Hisat2 v2.0.1 (Kim et al., 2015) then assembled and quantified using StringTie v1.2.2 software (Pertea et al., 2015). Gene expression levels were calculated as Transcripts Per Kilobase Million (TPM). Differentially expressed genes were analyzed with the R package of Ballgown (Frazee et al., 2015). Transcriptome analysis was performed according to a previously described transcriptome protocol (Pertea et al., 2016).

## Results

### Identification of *FT* genes in angiosperms

A genome-wide BLAST search for *FT* homologous genes was performed using the *Arabidopsis thaliana FT* (*AT1G65480*) protein sequence as a query against the database of 47 flowering plant transcriptomes, including two early angiosperms, 17 monocots, and 28 eudicots (**Table 1**). Considering that *FT* proteins share a high sequence similarity with other PEBP subfamilies, such as *MFT* and *TFL1* all containing the typical PEBP domain (Pfam database accession: PF01161), the BLAST hits may include genes from all three PEBP subfamilies. We therefore kept all BLAST hits containing the PEBP domain and longer than 100 amino acids. In total, 572 PEBP genes were identified from 47 plant species (**Table 1**) (350 genes from the monocot species) and were used for phylogenetic tree reconstruction. The phylogenetic tree showed that sequences from each of the three PEBP subfamilies, *MFT*, *TFL1*, and *FT*, formed a monophyletic clade (**Supplementary Figure 1**). For the *MFT* and *TFL1* subfamilies, there was no remarkable difference regarding gene numbers between monocot and eudicot species (**Table 1**, **Supplementary Figure 1**). However, the mean number of genes in the *FT* subfamily in each monocot genome is 14, whereas this number is only 3 in each eudicot genome.

**Table 1:** Numbers of *FT*, *TFL1*, and *MFT* genes in each angiosperm species.

Angiosperm	Abbr.	*FT*-like	TFL1-like	M*FT*-like	All	Reference
Early angiosperm						
***Nymphaea colorata***	Na	5	3	1	9	This study
***Amborella trichopoda***	Atr	1	1	1	3	This study
Monocots						
***Zostera muelleri***	Zmu	12	5	1	18	This study
***Zostera marina*** ***Zostera muelleri*** ***Spirodela polyrhiza***	Zma Zmu Sp	10 13 5	2 5 1	1 2 2	13 20 8	This study
***Phalaenopsis equestris***	Pe	7	0	1	8	(Hou and Yang, 2009)
***Musa acuminata***	Ma	14	5	3	22	This study
***Allium cepa***	Ace	7	NA	NA		(Lee et al., 2013a)
***Ananas comosus***	Aco	8	2	1	11	(Lv et al., 2012)
***Phoenix dactylifera***	Pd	5	1	1	7	This study
***Zea mays***	Zcn	15	6	3	24	(Danilevskaya et al., 2008)
***Sorghum bicolor***	Sb	13	4	2	19	(Wolabu et al., 2016)
***Setaria italica***	Si	11	4	2	17	This study
***Panicum virgatum***	Pv	27	7	6	40	(Grabowski et al., 2017)
***Panicum hallii***	Ph	15	4	2	21	This study
***Oryza sativa***	Os	13	4	2	19	(Itoh et al., 2010)
***Brachypodium distachyon***	Bd	12	3	3	18	(Wu et al., 2013)
***Aegilops tauschii***	Ata	19	3	2	24	This study
***Hordeum vulgare***	Hv	13	3	3	19	(Faure et al., 2007)
***Triticum aestivum***	Ta	46	9	8	63	(Halliwell et al., 2016)
Eudicots						
***Aquilegia coerulea***	Ac	3	4	1	8	This study
***Nelumbo nucifera***	Nn	7	4	2	13	This study
***Beta vulgaris***	Bv	2	2	0	4	(Pin et al., 2010)
***Solanum tuberosum***	St	3	4	1	8	(Navarro et al., 2011)
***Solanum lycopersicum***	Sl	5	5	1	11	(Lifschitz et al., 2006)
***Vitis vinifera***	Vv	1	3	1	5	(Carmona et al., 2007)
***Fragaria vesca***	Fv	2	3	1	6	This study
***Malus domestica***	Md	1	6	4	11	(Kotoda et al., 2010)
***Pyrus bretschneideri***	Pb	1	6	3	10	This study
***Prunus mume***	Pm	1	3	1	5	(Esumi et al., 2009)
***Cajanus cajan***	Cca	5	4	1	10	This study
***Glycine max***	Gm	13	8	2	23	(Wang et al., 2015)
***Phaseolus vulgaris***	Pvu	5	4	1	10	This study
***Medicago truncatula***	Mt	6	4	2	12	(Putterill et al., 2013)
***Populus trichocarpa***	Pt	2	3	1	6	(Hsu et al., 2011)
***Linum usitatissimum***	Lu	2	6	0	8	This study
***Cucumis sativus***	Cs	1	4	1	6	(Lin et al., 2007)
***Citrus clementina***	Ccl	2	3	1	6	This study
***Carica papaya***	Cp	1	3	1	5	This study
***Theobroma cacao***	Tc	1	3	1	5	This study
***Gossypium raimondii***	Gr	1	5	1	7	(Zhang et al., 2016)
***Thellungiella halophila***	Th	3	4	1	8	This study
***Thellungiella parvula***	Tp	3	2	1	6	This study
***Capsella rubella***	Cr	2	3	1	6	This study
***Brassica oleracea***	Bo	4	7	2	13	This study
***Lepidium meyenii***	Lm	4	0	4	8	This study
***Arabidopsis thaliana***	At	2	3	1	6	(Wang et al., 2015)
***Arabidopsis lyrata***	Al	2	4	1	7	This study

The numbers of FT, TFL1, and MFT proteins found in the genome of each species is indicated, especially in the FT gene family huge differences has shown between monocots and eudicots.

### Monocot *FT* genes form three clades and exhibit a high retention rate after gene duplication in grass

**Figure 2:**
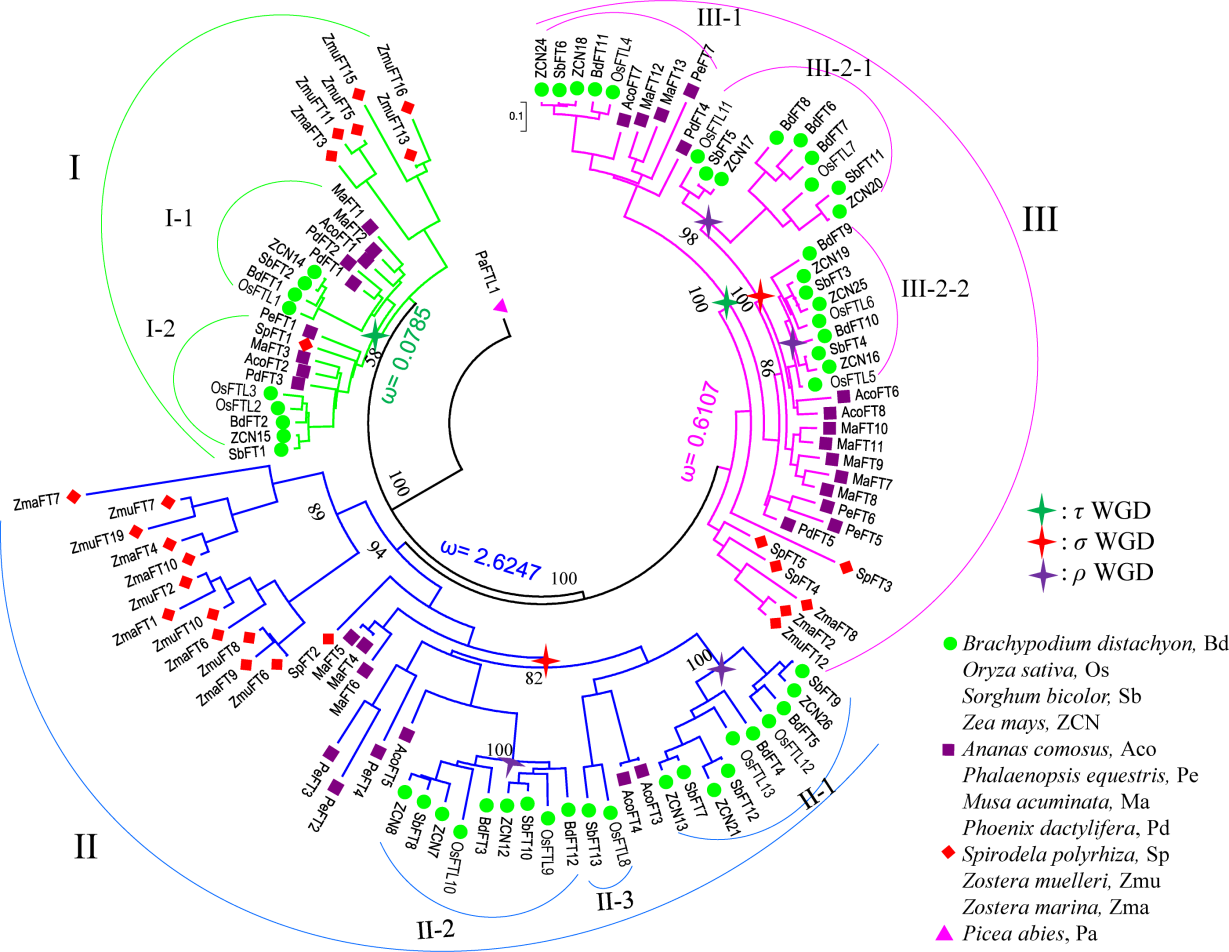
The phylogenetic relationship of the *FT* genes in monocots. The phylogenetic tree was constructed using the Bayes methods. The *P. abies FT*-like gene (JN039333) was set as the outgroup. The *FT* members from monocots are classified into three clades based on the tree; clade I is marked in green, II in blue, and III in pink. The stars mark the WGD event that occurred in monocots: green star, τ WGD; red star, ρ WGD; and purple star, σ WGD. Pa, *P. abies;* Zma, *Zostera marina*; Zmu, *Zostera muelleri*; Sp, *Spirodela polyrhiza*; Pd, *Phoenix dactylifera*; Ma, *Musa acuminata*; Pe, *Phalaenopsis equestris*; Aco, *Ananas comosus;* Bd, *Brachypodium distachyon*; Sb, *Sorghum bicolor*; Os, *Oryza sativa*; ZCN, *Zea mays.*.

To further explore the complex evolutionary history of the *FT* gene subfamily in angiosperms, all *FT* genes from the previous step and *FT* genes blasted from 1,000 plant transcriptomes were used to construct a maximum likelihood (ML) tree (**Figure 1**). Only one monophyletic group was identified in early angiosperms, magnoliids, Caryophyllales, ranunculids, Santalales, Saxifragales, asterids, and core rosids; however, there were three clades identified in monocots, suggesting that whole-genome duplication occurred in ancient monocots. Moreover, we constructed a Bayesian (Bayes) tree with all monocot *FT* protein sequences using the gymnosperm *Picea abies FT*-like gene as the outgroup (**Supplemental Table 2**, **Figure 2**). Based on the tree topology and posterior probability (> 0.8), the *FT* gene family in monocots could be classified into three clades, hereafter referred to as I, II, and III (**Figure 2**). Each monocot species examined, including early monocot seagrass (*Zostera muelleri* and *Zostera marina*) and *Spirodela polyrhiza*, and crown branches *Phoenix dactylifera*, *Phalaenopsis equestris*, pineapple, and all grass species, contained at least one *FT* copy in all three *FT* clades. In each of the three clades, seagrass and banana had more *FT* genes than the other monocots except Poaceae species. *Triticum aestivum* (*N* = 63) and *Panicum virgatum* (*N* = 40) had the largest number of *FT* genes among monocot species.

For clades I and III, all *FT* genes can be further divided into two subclades, I-1, I-2, and III-1, III-2, respectively, except the genes in early monocot seagrass (**Figure 2**). High confidence of the tree topology shown in **Figure 2** revealed that those subclades generated from an ancient duplication, which occurred after the split of *S. polyrhiza* and the other core monocots. In *FT* clade II, using pineapple *FTs* as the outgroup, it could be further divided into three subclades II-1, II-2, and II-3. Subclade II-3 contained only rice and sorghum *FT* genes (**Figure 2**). At least one copy of *FT* genes from pineapple and grass species was present in each of the II-1 and II-2 groups, suggesting that the two groups could be resulted from the σ WGD, which occurred 95~115 million years ago (MYA) before the split between grasses and pineapple (Tang et al., 2010; Ming et al., 2015). We found that, for several gene pairs in the II-1 and II-2 groups, the paralogs were located in syntenic regions in the rice and sorghum genomes (**Supplementary Figures 2A, B**), further supporting that the duplicates probably resulted from the σ WGD in grasses. In monocots, after the σ WGD, another WGD named ρ occurred 50~70 MYA (Paterson et al., 2004). Genes in clades II-1 and II-2 were thus further expanded by the ρ WGD, resulting in II-1a and II-1b, and II-2c and II-2d, respectively. Syntenic regions were also visible for these subclades (**Supplementary Figures 2C, D**). A similar expansion (**Supplementary Figures 2E, F**) might have also occurred for clade III-2, where the σ WGD occurred firstly, followed by a grass-specific ρ WGD event, leading to four subclades III-2-1a, III-2-1b, III-2-2c, and III-2-2d.

### Positive selection analysis

Given the possible functional divergence among the *FT* paralogs in the monocots identified above, we hypothesized that some of the *FT* (sub)clades might have undergone positive selection. To test this hypothesis, we used selection models implemented in the program *codeml* in the PAML package (Yang, 2007). Using branch models, our results showed that the three clades evolved under different selection pressures (*P* = 0.00398) (**Table 2**). Clades I and II had the lowest (0.0785) and highest (2.264) ω values (the ratio of non-synonymous to synonymous substitution rates), respectively, with clade III being in the middle (0.6107).

**Table 2:** Branch model tests for positive selection in monocots’ *FT* genes.

Model	LnL	Parameter estimates ω			P-value
		I	II	III	
Model 2	-20919.68	0.0785	2.264	0.6107	0.00398**
Model 0	-20926.34	0.1507			

LnL, log-likelihood value.

**Figure 3:**
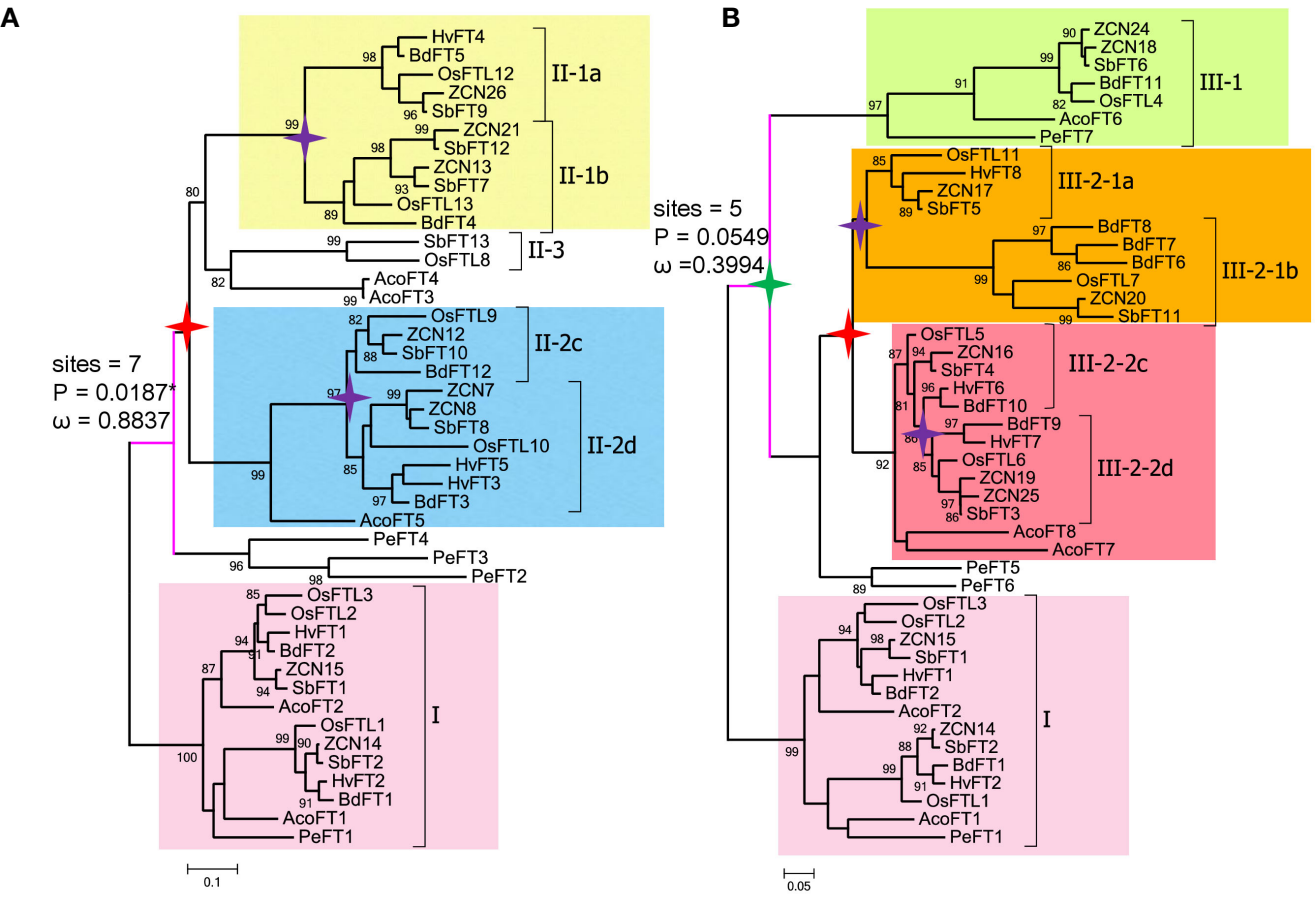
Pruned trees consisting of clade I and II (or III) and branch and branch site model tests. **(A)** Branch and branch site test using clade I as background and clade II as foreground. **(B)** Branch and branch site test using clade I as background and clade III as foreground. Green star, τ WGD; red star, ρ WGD; and purple star, σ WGD. ω denotes *dN*/*dS* values; sites indicate positive selection sites, P means p-value (probability). Digits in the branches are bootstrap values.

The branch site model is mainly used to detect the presence of positive selection sites on a clade. This method considers that the target branch has an omega value and all other branches share the same omega value. The branch model is mainly used to detect whether the omega value of the target branch is significantly higher than that of the background branch, that is, the gene evolution rate is accelerated on the target branch. Then, both branch and branch site models are employed to identify positively selected sites in each clade (**Tables 3**, **4**, **Figure 3**). In **Figure 3**, when setting clade II as the foreground and I and II as the background, clade II showed a high ω value with 0.8837; branch site selective, i.e., model A (allowing some sites in the foreground with ω >1), and null models were statistically different (*P* = 0.0187). Between clades I and II, the posterior probabilities for sites Q34, M155, and W138 that experienced positive selection were 0.965*, 0.943, and 0.910, respectively. The branch model showed a ω value with 0.3994 in clade III, when setting I and III as background. Positively selected sites between I and III were also evaluated. However, the null and selective models were not significantly different (*P* = 0.0549), and site L82 with the posterior probability 0.966 was identified in clade III.

**Table 3:** Amino acid sites under positive selection shown in the Ⅱ clade, when Ⅰ and Ⅱ were set as background.

Model	LnL	Parameter estimates					Positive selection sites	P-value
Model A	-14002.6589	Site class	0	1	2a	2b	33G 0.615 44R 0.570 34Q 0.965* 115M 0.943 120V 0.518 138W 0.910 150I 0.519	0.0187 *
		f	0.78710	0.13678	0.06485	0.01127		
		ω0	0.12773	1.00000	0.12773	1.00000		
		ω1	0.12773	1.00000	11.4757	11.4757		
Model A null	-14005.4222	1						

LnL, log-likelihood value.

**Table 4:** Amino acid sites under positive selection shown in the Ⅲ clade, when Ⅰ and Ⅲ were set as background.

Model	LnL	Parameter estimates					Positive selection sites	P-value
Model A	-14558.6940	Site class	0	1	2a	2b	25S 0.658 34Q 0.767 82L0.966* 103N 0.597 115A 0.769	0.0549
		f	0.85516	0.10245	0.03786	0.00454		
		ω0	0.10235	1.00000	0.10235	1.00000		
		ω1	0.10235	1.00000	998.887	998.887		
Model A null	-14560.5367	1						

LnL, log-likelihood value.

Within clades I and III, two subclades were generated from an ancient duplication. In clade I, using each of the two grass species as the foreground, we found relatively low ω values in each subclade by branch models (**Supplementary Figure 3A**). However, the branch site model showed that each subclade I-1 and I-2 had a highly statistically significant value and we identify five and six positively selected sites, respectively (**Supplementary Figure 3A**, **Table 5**). In clade III, each of two subclades III-1 and III-2 were set as the foreground separately, and we found no sites under positive selection (*P* = 1).

**Table 5:** Likelihood ratio test (LRT) of branch site models for the clade I.

Clades	Model	LnL	Parameter estimates					Positive selection sites	P-value
I-1	Model A	-5468.8904	Site class	0	1	2a	2b	15V0.542 36E 0.946 47Q 0.929 115A 0.623 168S 0.655	0.0128*
			f	0.91440	0.06267	0.02145	0.00147		
			ω0	0.05936	1.00000	0.05936	1.00000		
			ω1	0.05936	1.00000	43.9035	43.9035		
	Model A null	-5471.9865	1						
I-2	Model A	-5468.5275	Site class	0	1	2a	2b	25S 0.518 26I 0.916 44R0.695 47Q 0.935 103N0.981* 155L 0.502	0.0242*
			f	0.89845	0.06521	0.03387	0.00246		
			ω0	0.05847	1.00000	0.05847	1.00000		
			ω1	0.05847	1.00000	12.8907	12.8907		
	Model A null	-5471.0687	1						

LnL, log-likelihood value.

Within clades II and III-2, σ WGD and ρ WGDs had occurred and formed four subclades. Clade II formed II-1a, II-1b, II-2c, and II-2d after experiencing σ WGD and ρ WGD events. Clade III-2 formed III-2-1a, III-2-1b, III-2-2c, and III-2-2d after experiencing σ WGD and ρ WGD events (**Figures 3A, B**). To determine selection types and identify corresponding sites in II and III-2 clades, two types of tests were used. Using each of the subclades in clade II as a foreground branch and the remaining subclades as the background, the ω values ranged from 0.1305 to 0.3727 (**Supplemental Table 3**), indicating that clade II was conserved. Positively selected sites were only identified in subclades II-1a (R107 and T144 with posterior probabilities being 0.609 and 0.938, respectively) and II-1b (T91 with probabilities being 0.552) (**Supplementary Figure 3B**, **Supplemental Table 4**). Subsequently, we tested the positive selection in the II-1a subclade. Six positively selected sites were identified in the II-1a subclade, indicating that the II-1a subclade had the fastest divergence rate in II (**Supplemental Table 5**). Similar tests were performed in the III-2 clade; the ω value varied among subclades: 0.0001 for III-2a, 0.1015 for III-2b, 0.0761 for III-2c, and 0.1514 for III-2d (**Supplemental Table 6**). Using the branch site model, only III-2-2d (four sites R35, L61, T96, and E160) and III-2-2c (two sites Q49 and E55) had positive selection sites, although not statistically significant (**Supplementary Figure 3C**, **Supplemental Table 7**).

### Conservation and diversification in sequence composition

Based on the present model, the *FT* protein was induced by the photoperiod pathway and transported from leaf to the shoot apex, where it interacted with intracellular receptors, 14-3-3 proteins, to form a complex that can bind an FD transcription factor. They formed a “florigen activation complex” (FAC) to activate the expression of the floral-meristem genes (Taoka et al., 2011). There were three important regions for *FT* genes. The first was the region-binding 14-3-3 proteins (Taoka et al., 2011). The second was the region-binding chemical ligands (Y85) (Hanzawa et al., 2005). The third was a divergent external loop (Ahn et al., 2006). The latter two regions might be vital for the activity difference between *FT* and *TFL1* genes in *Arabidopsis thaliana*. Our analysis focused on these regions as they were more likely to affect functions.

**Figure 4:**
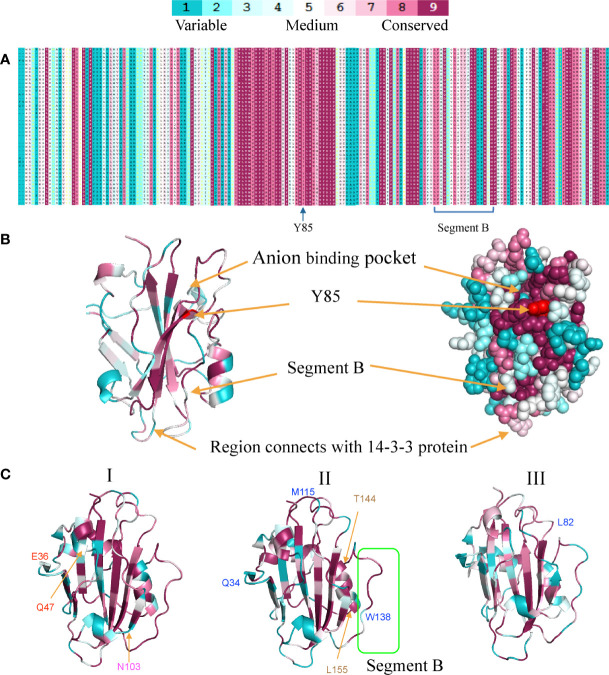
Sequence conservation and variation among three *FT* clades in monocots. **(A)**. Multiple-sequence alignment of the *FT* gene family. **(B)** Homology modeling of the *FT* gene family shows the conservation degrees (displayed in different colors) of different regions. **(C)** Conservation differences among three monocot *FT* gene clades using ribbon diagrams. Positive selection sites (BEB value >0.9) also marked in three clades. All the figures were generated using the PyMol script with the output of ConSurf as input. Blue marks represent positive selection sites with clade I Red marks represent positive selection sites in clade I-1, magenta marks represent positive selection sites in clade I-2, and coffee marks represent positive selection sites in clade II-1a.

**Figure 5:**
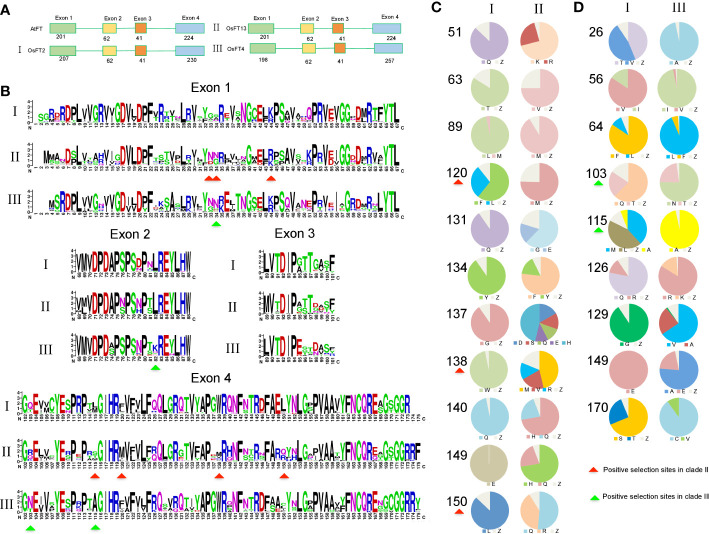
Characterization of sequences in three *FT* clades. **(A)** Exon and intron structures among three *FT* clades. **(B)** Logos representing motifs conserved within three clades. **(C)** Frequency of candidate amino acid residues responsible for function divergence between clade I vs. II. **(D)** Frequency of candidate amino acid residues responsible for function divergence between I vs. III. Red arrows represent positive selection sites in clade II, and green arrows represent positive selection sites in clade III.

Based on the homolog model of Arabidopsis *FT* (PDB ID: 1WKP) and the multiple-sequence alignment of monocot *FT* proteins, we calculated the conservation score of each position using the ConSurf web server (Ashkenazy et al., 2016). We found that the region around the anion binding pocket and Y85 are highly conserved among the *FT* proteins (**Figures 4**, **5**). However, segment B and the region interacting with the 14-3-3 protein were variable, suggesting that the *FT* genes in monocots might have undergone functional divergence. We also used ConSurf for each of the three clades. We found that segment B was highly conserved in clade I but were variable at some sites in clades II and III, especially W138 in the segment B region which was also a positive selective site for clade II. The region interacting with 14-3-3 proteins was highly variable in clade II but strictly conserved in clades I and III (**Figure 5**).

Amino acid changes in the PEBP gene family could lead to important functional changes. For example, a change of one amino acid (Y85H) results in function conversion from *FT* to *TFL1*(Hanzawa et al., 2005). To identify important sequence changes that led to the function divergence of the *FT* genes in monocots, we checked amino acid substitutions in each clade. Using the rice gene *Heading date 3a* (Os*FT*2) as reference, we identified that 63 and 53 amino acids were highly variable between clades I and II and between clades I and III, respectively (**Figures 4A, B**). Based on a previous study about *FT* mutants in Arabidopsis (Ho and Weigel, 2014), about 50% of these amino acid changes were predicted to affect functions, suggesting that these variable sites might be important for the function diversification of monocot *FT* genes. In our study, 11 and 9 amino acid sites showed high difference between clades I and II and between I and III, respectively (**Figures 4C, D**). Among them, amino acid residues 120, 138, and 150 were positive selection sites in clade II and amino acid residues 103 and 115 were positive selection sites in clade III simultaneously.

To further estimate the functional diversification (FD) between two paralogs, we used the software DIVERGE 3.0 (Gu, 2006). Given amino acid sequence alignments, DIVERGE could predict functional changes among paralogs. The software includes two models, FD I and FD II, based on the evolutionary rates and biochemical properties of amino acids (Gu, 1999). Between clades I and II, seven putative sites (13, 16, 39, 53, 81, 138, and 149) responsible for FD were identified (**Supplementary Tables 8**, **9**). These amino acid sites were highly conserved in clade I but different between clades I and II, particularly sites 81, 138, and 149. Meanwhile, sites 138 and 149 were also positive selection sites in clade II (**Figure 4C**, **Supplementary Figure 4**). Notably, site 138 was located in the segment B region. In addition, 18 and 31 sites were identified for the gene pairs in clades I and III and clades II and III, respectively. In clades I and III, sites 26 and 115 were also positive selection sites in clade III; at the same time, sites 97 and 141 were located in a region involving an interaction between 14-3-3 protein and segment B (**Supplementary Figure 5**). Next, we examined the paralog pairs generated from several duplications. In III-1 and III-2, we found that three sites (13, 25, and 45) might be responsible for functional divergence. In the σ WGDs, three (16, 45, 150) and two (132, 136) positions were identified when comparing II-1 and II-2 and comparing III-2-1 and III-2-2, respectively, including one (position: 150) that was functionally important in soybean *FT* genes and two (positions: 132 and 136) located in segment B.

### Expression profiles of different clade *FT* genes in rice and maize

**Figure 6:**
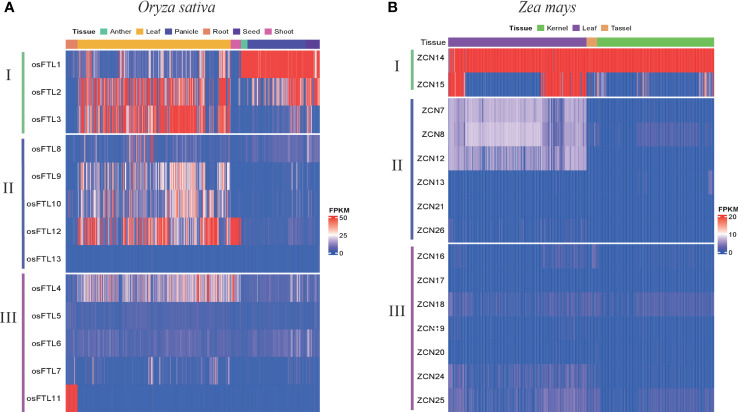
The expression patterns of the three clades’ *FT* gene in two monocot species including **(A)** *Oryza sativa* and **(B)** *Zea mays*.

To further characterize whether different clade genes possess differential expression patterns in monocots, we investigated expression patterns in two representative monocot plants, *Oryza sativa* and *Zea mays*. Transcriptome data were downloaded from the Plant Public RNA-seq Database (PPRD, http://ipf.sustech.edu.cn/pub/plantrna/) (Yu et al., 2022), providing a large number of RNA-seq libraries, 19,664 and 11,726 for maize and rice, respectively. Generally, clade I genes in both rice and maize exhibited higher expression levels than clade II and III genes in all studied organs and tissues. For rice, *OsFTL1*, *OsFTL2*, and *OsFTL3* displayed high expression patterns across tissues, including leaf, shoot, flower, anther, panicle, seed, and endosperm (**Figure 6A** and **Supplementary Figure 6A**). Although in the same clade *OsFTL1* presented opposite expression patterns compared with *OsFTL2* and *OsFTL3*, *OsFTL2* and *OsFTL3* showed high expression only in leaf but *OsFTL1* presented high expression in all tissues except leaf and root. For clade II, three genes (*OsFTL9*, *OsFTL10*, and *OsFTL12*) in leaf and one gene (*OsFTL12*) in stem, shoot, and leaf were expressed at relatively high levels. *OsFTL8* was in a low expression level of transcripts in all stages and tissues, and *OsFTL13* expression was not detected in any organs. In clade III, it was likely that *OsFTL11* was expressed preferentially high in root, whereas other *FT* genes in the same clade or two former clades were barely expressed in this tissue, so *OsFTL11* was considered as a root-specific expressed gene. *OsFTL4* could be observed expressed in leaf and shoot, and the rest of the *FT* genes in clade III were in a very low level of transcripts in all tissues. A similar phenomenon can be seen in maize, with *ZCN14* and *ZCN15* showing stronger expression levels than the other two clades’ *FT* genes (**Figure 6B** and **Supplementary Figure 6B**), especially *ZCN14* which was expressed at a significantly higher level in all tissues and organs. *ZCN15* was strongly expressed in leaf, flower, and stem but not the same as ZCN14 which was expressed in a wide range of tissues. In clade II, *ZCN7*, *ZCN8*, and *ZCN12* presented a low level of transcripts only in leaf and nearly no expression in remaining tissues. More than that, *ZCN13*, *ZCN21*, and *ZCN26* were hardly expressed in all tissues. All clade III genes showed little or merely no expression in any tissues, so we speculated that this clade’s genes may already lose functions. Overall, different clade genes showed differing expression patterns and genes belonging to the same clade did not always have the same expression patterns. Among *FT* members, clade I *FT* genes showed the strongest transcript level, followed by clade II, and clade III exhibited the weakest expression patterns in almost all tissues. In addition, all expressed *FT* genes kept relatively high expression levels in leaf, a primary site of *FT* protein synthesis, which is consistent with a previous study that *FT* is primarily expressed in the leaves and the resulting protein travels to the meristem where it initiates a transition of the vegetative meristem to the floral meristem (Wong et al., 2013). These data supported that clade I *FT* genes were the most likely candidates to be involved in the regulation of flowering time in monocots.

To detect FT genes’ expression under vernalization treatment, RNA-seq was performed on 11 species including five monocots (*Allium sativum*, *Brachypodium distachyon*, *Dactylis glomerate*, *Hordeum vulgare*, and *Triticum aestivum*) and six eudicots (*Aquilegia coerulea*, *Arabidopsis thaliana*, *Brassica napus*, *Brassica rapa*, *Camelina sativa*, and *Mimulus guttatus*). During vernalization, higher *FT* gene transcripts were detected in monocots such as *A. sativum* (4.77 TPM), *B. distachyon* (33.03 TPM), *D. glomerate* (26.52 TPM), *H. vulgare* (23.25 TPM), and *T. aestivum* (4.08 TPM) but lower expression in eudicots *A. coerulea* (0.61 TPM), *A. thaliana* (0.12 TPM), *B. napus* (3.81 TPM), *B. rapa* (0.17 TPM), *C. sativa* (1.97 TPM), and *M. guttatus* (0.27 TPM) (**Supplementary Figure 7A**). Comparing vernalization and non-vernalization circumstances, one clade III *FT* gene in *B. distachyon*, one clade II *FT* gene in *D. glomerate*, two clade I *FT* genes in *H. vulgare*, and one clade II *FT* gene in *T. aestivum* showed a huge difference (**Supplementary Figures 7B–E**).

### Co-expression analysis of *FT* genes in rice and maize

Duplicated genes generated from tandem and segmental duplications have multiple fates. Some genes maintain their ancestral functions, whereas others may be pseudogenized or neofunctionalized (Birchler and Yang, 2022). Combining the co-expression network from PPRD with rice co-expression modules that associated with agronomic traits (Yu et al., 2022; Zhang et al., 2022), we further surveyed *FT* genes’ function in different clades.

**Figure 7:**
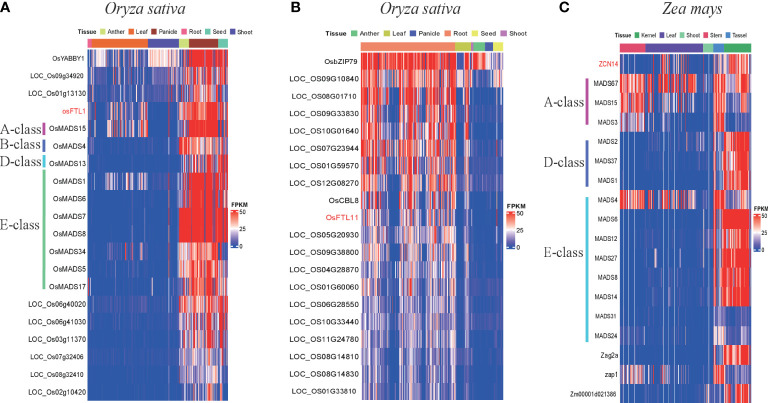
Expression patterns of *FT* and their co-expression genes in *Oryza sativa* and *Zea mays*. **(A)** Expression patterns of *OsFTL1* and its co-expression genes in anther, leaf, panicle, root, seed, and shoot; different MADS-box classes labeled in different color lines. **(B)** Expression patterns of *osFTL*11 and its co-expression genes in anther, leaf, panicle, root, seed and shoot; different MADS-box class labeled in different color line. **(C)** Expression patterns of *ZCN14* and its co-expression genes in kernel, leaf, shoot, stem, and tassel; different MADS-box classes labeled in different color lines.

For rice, *OsFTL1* in clade I was presented in M0166 whose module correlated with flower development traits (**Table 6**). Ten floral development-related MADS-box genes had similar expression patterns with *OsFTL1*, such as *OsMADS15* (A-class genes), *OsMADS4* (B-class genes), *OsMADS13* (D-class genes), *OsMADS1*, *OsMADS5*, *OsMADS6*, *OsMADS7*, *OsMADS8*, *OsMADS17*, and *OsMADS34* (E-class genes) (**Figure 7A** and **Supplementary Figure 8A**), and they were expressed in panicle, flower, anther, pistil, seed, and endosperm and especially highly in floral organs and tissues. Among these MADS-box genes, *OsMADS7*, *OsMADS8*, and *OsMADS15* had been validated to regulate flowering time (Cui et al., 2010; Lu et al., 2012). These results indicated that *OsFTL1* might modulate both floral organ development and flowering time in rice. *OsFTL2*, *OsFTL3*, *OsFTL9*, and *OsFTL10* exhibited leaf-preferred expression patterns; these genes were in M0166 whose module is related to flowering time traits. *OsFTL11* was expressed mainly in root and expressed hardly in other organs. Three NB-ARC genes (*LOC_Os10g33440*, *LOC_Os08g14830*, and *LOC_Os08g14810*), *LOC_Os01g33810* (disease resistance protein RPM1), and *OsCBL8* had similar co-expression patterns with *OsFTL11*; these genes were more or less associated with disease resistance, suggesting that *OsFTL11* might acquire a new function of responding to stress (**Figure 7B** and **Supplementary Figure 8B**). *OsFTL4* in clade III and *FTL8* in clade III co-expression modules were correlated with aluminum sensitivity and stress resistance, respectively (**Table 6**). In addition to the *FT* genes mentioned above, other *FT* genes in rice either cannot elucidate a clear function or were in a low expression level.

**Table 6:** Rice *FT* co-expression module information.

Gene name	Clade	Predicted trait	Modules
*FTL1*;	I-1	Flower development trait;	M0166
*FTL*2;*FTL*3;*FTL9*;*FTL10*;	I-2, I-2, II-2, II-2	Flowering time;	M0166
*FTL8*	II-3	Biotic and abiotic stress resistance	M0042
*FTL4*	III-1	Aluminum sensitivity	M0032
*FTL11*	III-2-1	Disease resistance	M0126;M0473
*FTL6*	III-2-2	–	M0062;M0490
*FTL12*	II-1	–	M0062
*FTL7*	III-2-1	–	M0164

Modules data collect from Yu Zhang et al. (Zhang et al., 2022); - represents uncertain trait.

In maize, *ZCN14* was highly expressed in all tissues and was in the same co-expression network with 14 MADS-box genes, comprising three A-class genes (MADS3, MADS15, and MADS67), three D-class genes (MADS1, MADS2, MADS37) and eight E-class genes (*MADS4*, *MADS6*, *MADS12*, *MADS27*, *MADS8, MADS14*, *MADS24*, and *MADS31*) except B-class genes and C-class genes (**Figure 7C** and **Supplementary Figure 8C**). Similar to rice, those MADS-box genes were also expressed mainly in reproductive organs and tissues (flower, anther, pistil, and kernel). In addition, C-class MADS-box genes were absent in both the rice and maize clade I co-expression network, suggesting that *OsFTL1* and *ZCN14* might not have participated in pistil formation. *ZCN17*, a maize clade III-2-1 *FT* gene, was slightly and exclusively expressed in root. So far, we found that both *ZCN17* and *OsFTL11* presented root-specific expression patterns; moreover, they were all in clade III-2-1, so *ZCN17* may also play roles in responding to disease. These results suggest that clade III-2-1 *FT* genes in Poaceae might already possess this function and their descendant genes inherited it.

These results indicated that *FT* genes in clade I might retain their ancestral function of activating flowering and some of clade I *FT* genes also played an essential role in regulating floral organ development. In clades II and III, *FT* genes had various probabilities: losing functions, acquiring new functions, or regulating flowering time.

## Discussion

### Evolutionary history of monocot *FT* genes

Studies on individual genomes suggested that the numbers of *FT* genes varied among species. For instance, only *FT*-like genes but not florigen-functioning *FT* genes were found in gymnosperms (Klintenäs et al., 2012). Only one *FT* gene was detected in *Amborella trichopoda*(Drabešová et al., 2016), two *FT* genes were found in *Arabidopsis thaliana*(Wang et al., 2015), and 13 *FT* genes were found in rice (Tamaki et al., 2007). Previous studies revealed that rice and other cereals contained eight common ancestors for *FT*-like genes (Chardon and Damerval, 2005). Adding more genomes and the 1KP database, our results extended the previous observations and revealed that the gene numbers were systematically different between monocots, eudicots, and magnoliids, only one clade for early angiosperm, magnoliids, and eudicots but three for monocots. This variation might be caused by a series of WGDs followed by differential gene retention and loss rates between eudicots and monocots, like the *SET* and JmjC domain gene families (Zhang and Ma, 2012; Qian et al., 2015). Although eudicots have also experienced a series of WGDs (e.g., α, β, and γ in *Arabidopsis*(Bowers et al., 2003)), most duplicated *FTs* were probably lost, except for soybean and *Chenopodium quinoa*(**Supplementary Figure 6**, **Figure 7**). Monocots had also experienced three WGD events: τ, σ, and ρ (Tang et al., 2010; Lee et al., 2013b; Jiao et al., 2014), but lots of *FT* genes had been retained, especially in grasses. Such as in clades I and III, *FT* genes were divided into two subclades, I-1, I-2, and III-1, III-2, respectively. Those subclades did not include genes from early monocots *Z. marina* and *S. polyrhiza*. High confidence of the tree topology in **Figure 2** suggested that the subclades in I and III might be derived from the τ WGD (Jiao et al., 2014), which occurred after the split of *S. polyrhiza* and the other core monocots approximately 150 MYA. The other two WGDs σ and ρ occurred before and after the split between grasses and *A. comosus*(Tang et al., 2010; Ming et al., 2015). In our study using *Picea abies FT-like* (JN039333) as the outgroup, at least one copy of *FT* genes from pineapple and grass species were present in clades II-1 and II-2. Moreover, syntenic regions were identified in those clades, suggesting that the two groups could be resulted from the σ WGD, which occurred 95~115 MYA. Alternatively, syntenic regions of *FT* genes were found in II-1a and II-1b, and II-2c and II-2d, respectively, indicating further expansion by ρ WGD (Paterson et al., 2004). A similar expansion (one σ and two ρ WGDs) also happened in clade III-2, resulting in four subclades. All those results reveal that WGDs are the main reason for the *FT* gene expansion in monocots.

### Functional diversification of *FT* genes in monocots

In total, lots of *FT* genes in monocots provided abundant genetic materials for function diversity to face complex circumstances. For example, three FT genes (*SbFT1*, *SbFT8*, and *SbFT10*) were identified as florigen in sorghum (Wolabu et al., 2016), whereas *SbFT12* had the function to repress flowering (Cuevas et al., 2016). Under photoperiod and vernalization, *HvFT3* and *TaFT3* delayed flowering under short-day photoperiods in winter and induced flowering in long days in the spring (Halliwell et al., 2016). In our study, *FT* genes’ high expression in monocots but not eudicots repress flowering under vernalization. In *Allium cepa*, four *FT* genes with different functions including, repressing flower, bulbing, and bulb formation (Lee et al., 2013a).

Here, our results revealed that after the divergence of monocots and dicots, the common ancestor of monocots underwent ancient duplication events, giving rise to the three clades’ *FT* genes in monocots (Paterson et al., 2004; Tang et al., 2010; Ming et al., 2015), which we named clades I, II, and III. Subsequently in Poaceae, clade I *FT* genes were duplicated after experiencing τ WGD events and formed I-1 and I-2 subclades, clade II *FT* genes were duplicated twice after experiencing τ and σ WGD events, and duplicated *FT* gene pairs in clade III were retained after undergoing three rounds of WGD events (i.e., τ, σ, and ρ WGD events). *FT* genes in each clade evolved in its own way and thus probably functional differentiation arose during the subsequent evolutionary process.

Clade I *FT* genes are relatively conserved in terms of key amino acid sites and protein binding region, and large-scale transcriptome analysis of the rice and maize reveals that the clade I genes maintained high expression levels in almost all tissues, especially in leaves. We summarized almost all *FTs* with definite function in monocots (**Table 7**). These show that clade I might be the most conserved clade and represent the ancestral functions of *FT*, and the other two clades might have experienced functional diversification such as subfunctionalization, neofunctionalization, or loss of function. Based on the above results, we hypothesize that the clade I genes might be the most conserved genes to the ancestral *FT* genes, inheriting and maintaining the regulatory function of flowering time. Apart from that, some clade I genes were co-expressed with a large number of MADS-box genes, crucial genes for flower development, which suggested that clade I genes may also be involved in floral organ development in addition to mediating the flowering time in monocots.

**Table 7:** *FT* gene in each monocot species.

Species	Gene name	Clade	Function	Reference
Rice	*OsFTL2/Hd3a*	I	SD induction	(Kojima et al., 2002; Komiya et al., 2008; Komiya et al., 2009)
	*OsFTL3/RFT1*	I	LD induction	
Maize	*ZCN8*	II	SD induction	(Danilevskaya et al., 2008; Danilevskaya et al., 2010; Lazakis et al., 2011; Meng et al., 2011)
	*ZCN15*	I	Expression in kernel	
	*ZCN14*			
Sorghum	*SbFT12*	II	Repression	(Cuevas et al., 2016; Wolabu et al., 2016)
	*SbFT1*	I	Induction	
	*SBFT10*	II	Introduction	
	*SbFT8*	II	LD induction	
Barley	*HvFT1*	I	LD induction	(Faure et al., 2007; Kikuchi et al., 2009; Digel et al., 2015)
	*HvFT2*	I	LD induction	
	*HvFT3*	II	SD and LD induction	
Wheat	*TaFT1*	I	LD induction	(Li et al., 2015; Halliwell et al., 2016)
	*TaFT3*	II	SD induction	
*Brachypodium*	*BdFT1*	I	Induction	(Ream et al., 2014)
Distachyon	*BdFT2*			
Swichgrass	*PvFT8*	II	Induction	(Grabowski et al., 2017)
Pineapple	*AcoFT1*	I	Induction	(Lv et al., 2012)
*Phalaenopsis* orchid	*PeFT*	I	Induction	(Li et al., 2014)
Onion	*AceFT1*	I	Bulb formation	(Lee et al., 2013a; Manoharan et al., 2016)
	*AceFT2*	I	Induction	
	*AceFT4*	II	Repression	
	*AceFT7*	III	Onion bulbing	

Clade II genes experienced higher levels of positive selection pressure compared with the clade I gene. Seven positively selected sites (G33, Q34, R44, M115, V120, W138, and I150) were identified between clades I and II. Among those sites, three positive selection sites (W138, I150, and M115) were proved to be vital for florigen function in Arabidopsis, soybean, and other species, and notably W138 also acts as a functional divergence (FD) site which is an important site located at the segment B region (Ahn et al., 2006) and it has shown that flowering was repressed when W138 was mutated to R138 in Arabidopsis, sorghum, and onion (Lee et al., 2013a; Ho and Weigel, 2014; Cuevas et al., 2016). In addition, I150 has been replaced by R150, which is a vital site in soybean, in which I150 changed into E or Y with repression function (Ho and Weigel, 2014; Wang et al., 2015). Another substitution site 115, which induces weak florigen function in soybean (Wang et al., 2015), is also under positive selection. The region interacting with 14-3-3 proteins is also highly variable in clade II, with about half of the genes maintaining a low expression level and the other genes being largely unexpressed in all tested organs for rice and maize transcriptome analysis. Three clade II *FT* genes in *B. distachyon*, three clade II FT genes in *D. glomerate*, and one gene clade II FT in wheat showed high expression under vernalization circumstances to repress flowering in winter.

Similar to clade II, clade III had a relatively high positive selection pressure, segment B region was also variable, and only few genes lowly expressed or showed tissue-specific high expression patterns, which indicates that non-functionalization or neofunctionalization might have occurred in clade III. In **Figure 2**, clade III can be divided into two subclades III-1 and III-2. The expression of genes in III-1 had similar expression patterns with florigen functional genes but have extremely low expression in each tissue, which demonstrated that III-1 might keep a part of ancient *FT* gene function in controlling flowering time. Genes in III-2-2c have been replaced with a change from Y85 to N85 (**Supplementary Figure 5**). Given that Y85 is very important for florigen function (Hanzawa et al., 2005), the substitution in the subclade may result in acquisition of a new function or loss of the original function. Substitution in this site also occurred in some early monocot species changing from Y85 to H85 (Hanzawa et al., 2005), which have been proved in *Arabidopsis* to form *TFL1* function. In clade III-2, four positively selected sites have been detected in III-2-2d, among which sites 61 and 96 were located at the region interacting with the 14-3-3 protein. In this subclade, L61 was replaced by R or G, and T96 was replaced by G, which suggests that those genes in this subclade could not interact with the 14-3-3 protein nor the *FD* protein to promote flowering and thus are functionally diverged (Taoka et al., 2011). Genes in III-2-1a (*OsFTL11* in rice and *ZCN17* in maize) were highly expressed only in roots and *OsFTL11* was co-expressed with many disease-resistant R genes, so it is speculated that clade III genes may have diverged into a new function and be involved in disease resistance.

Our study revealed that after different rounds of WGD events, each clade or subclade gene underwent different levels of amino acid substitutions and were subjected to different selective pressures, which then led to functional divergence, so some genes maintained original functions, whereas other genes might have generated new functions or lost their original functions *via* the accumulation of amino acid mutations. The results from our study will provide highly valuable insights for future functional research of *FTs*.

## Conclusion

In summary, compared with the monophyletic group identified in early angiosperms, magnoliids, and eudicots, more *FT* genes were present in monocots and divided into three clades (I, II, and III). The whole-genome duplication was probably the main driver for increasing gene numbers in the *FT* gene family. Most of florigen-functioning *FT* genes had been identified in clade I, and all of them are under purifying selection and had a high expression level compared with clade II and III genes, suggesting that clade I represents the ancestor of *FT* genes in monocots. We found that II and III *FT* genes might be subject to positive selection. Our study highlighted that the role of positive selection, particularly the positively selected site substitutions in the conserved functional domain, might be the reason for gene functional divergence in *FT* genes. Taken together, our results revealed that the sequences of *FT* genes in monocots were variable under positive selection, which provides new insights into the mechanism of gene duplication, gene retention, and functional divergence to adjust the rapid changing environment.

## Data availability statement

The datasets presented in this study can be found in online repositories. The names of the repository/repositories and accession number(s) can be found in the article/**Supplementary Material**.

## Author contributions

LZ conceived and designed the study. XL analyzed sequence data and performed phylogenetic reconstructions with FC. HL conducted transcriptome analysis. HL, XL, and LZ wrote the manuscript with input from all authors.
