# Supplementary material for: Large-scale analyses of angiosperm Flowering Locus T genes reveal duplication and functional divergence in monocots
Source: Front Plant Sci. 2023 Jan 4;13:1039500. doi: 10.3389/fpls.2022.1039500 (PMC9847362; doi:10.3389/fpls.2022.1039500)
Supplement: Supplementary file 1 [file DataSheet_1.docx]

**
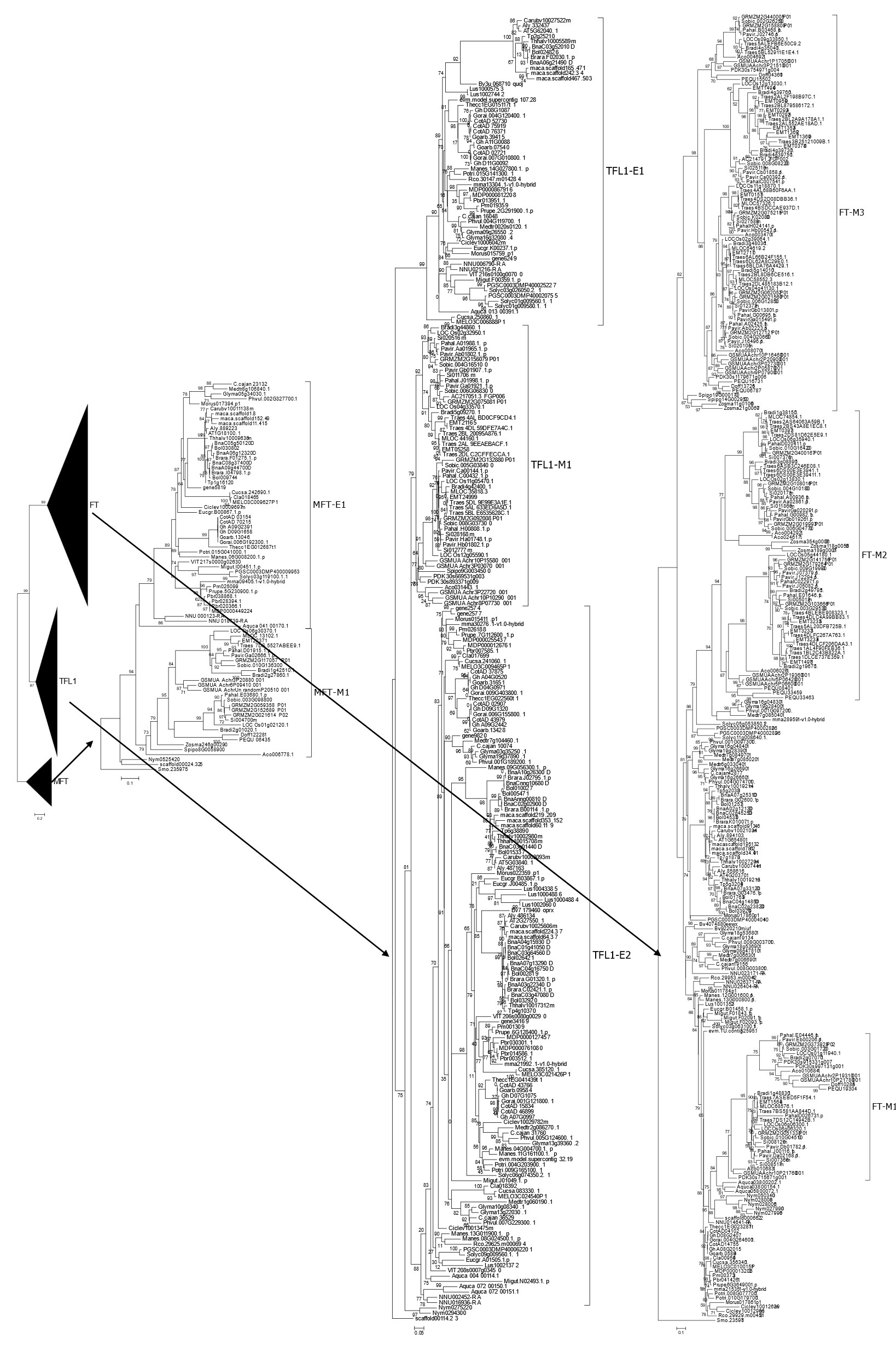
Supplementary Figure 1.** Three groups of *PEBP* gene family in angiosperm.


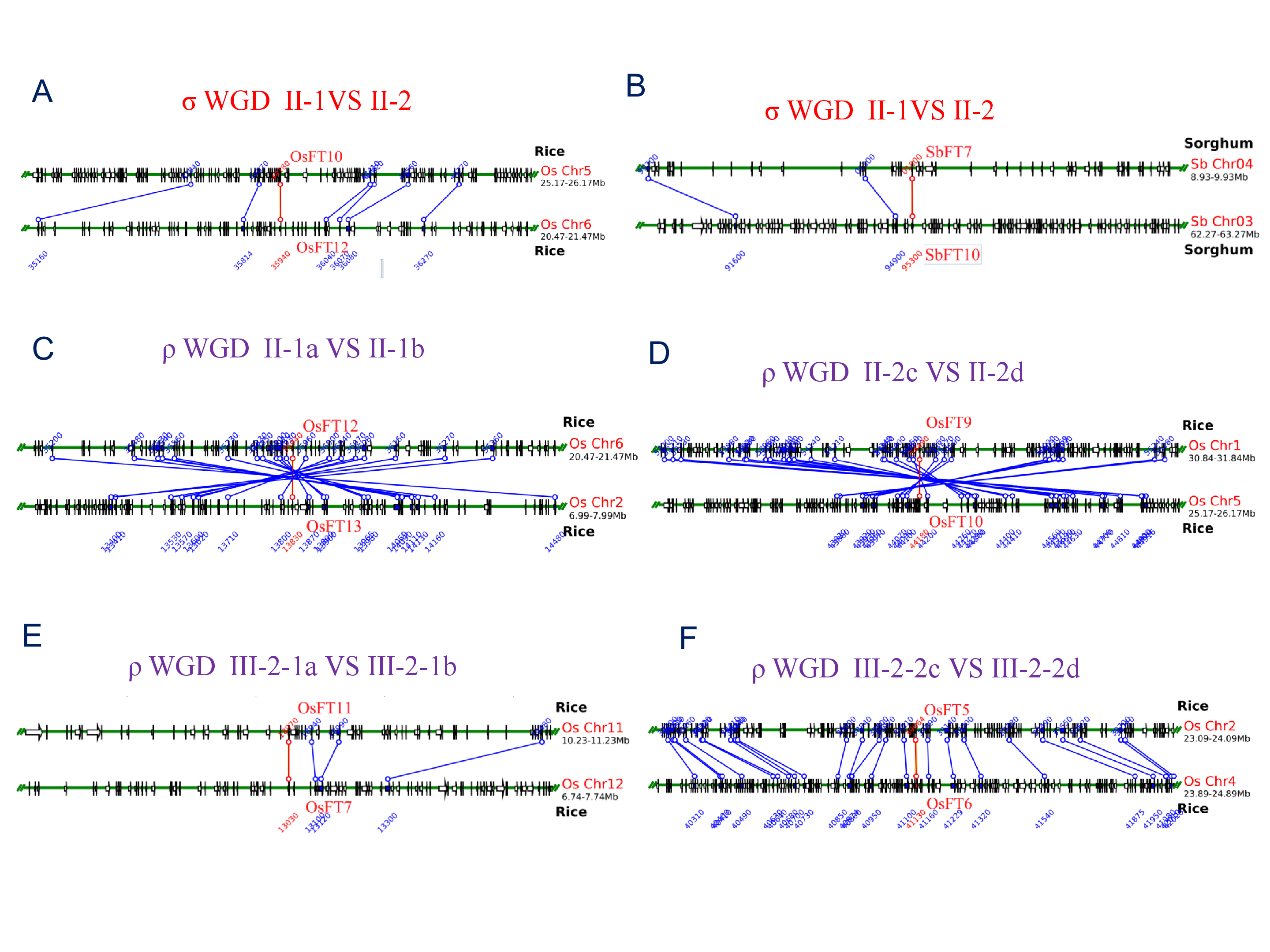


**Supplementary Figure 2.** Illustration of some grasses with the syntenic regions containing representative duplicated gene pairs from recent polyploidy events.

**
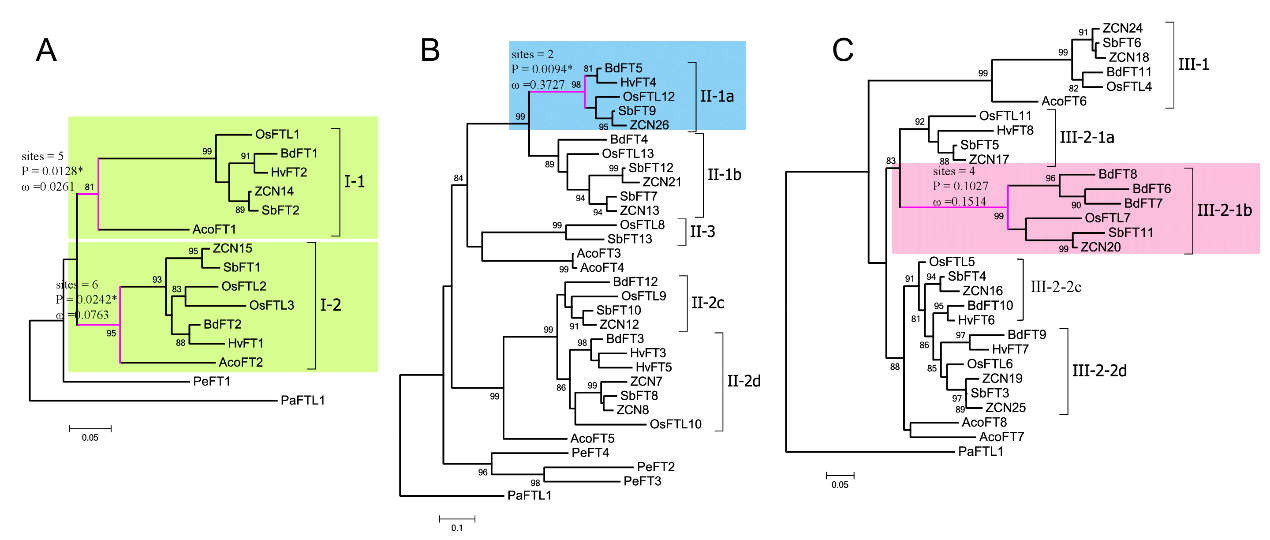
**

**Supplementary Figure 3.** Branch and branch site model tests within clade I, II, and III.

**
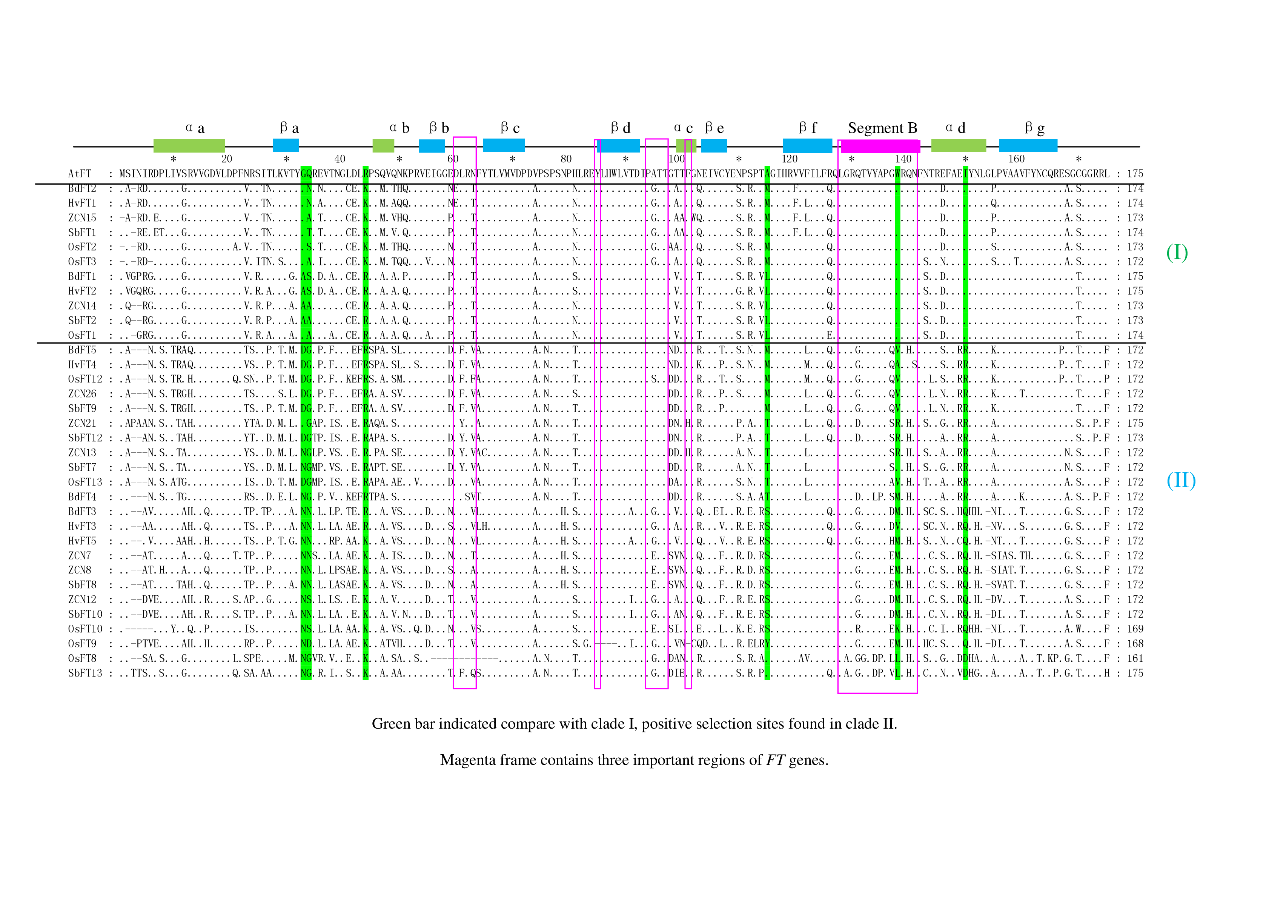
**

**Supplementary Figure 4.** Sequences alignment between I and II clades.

**
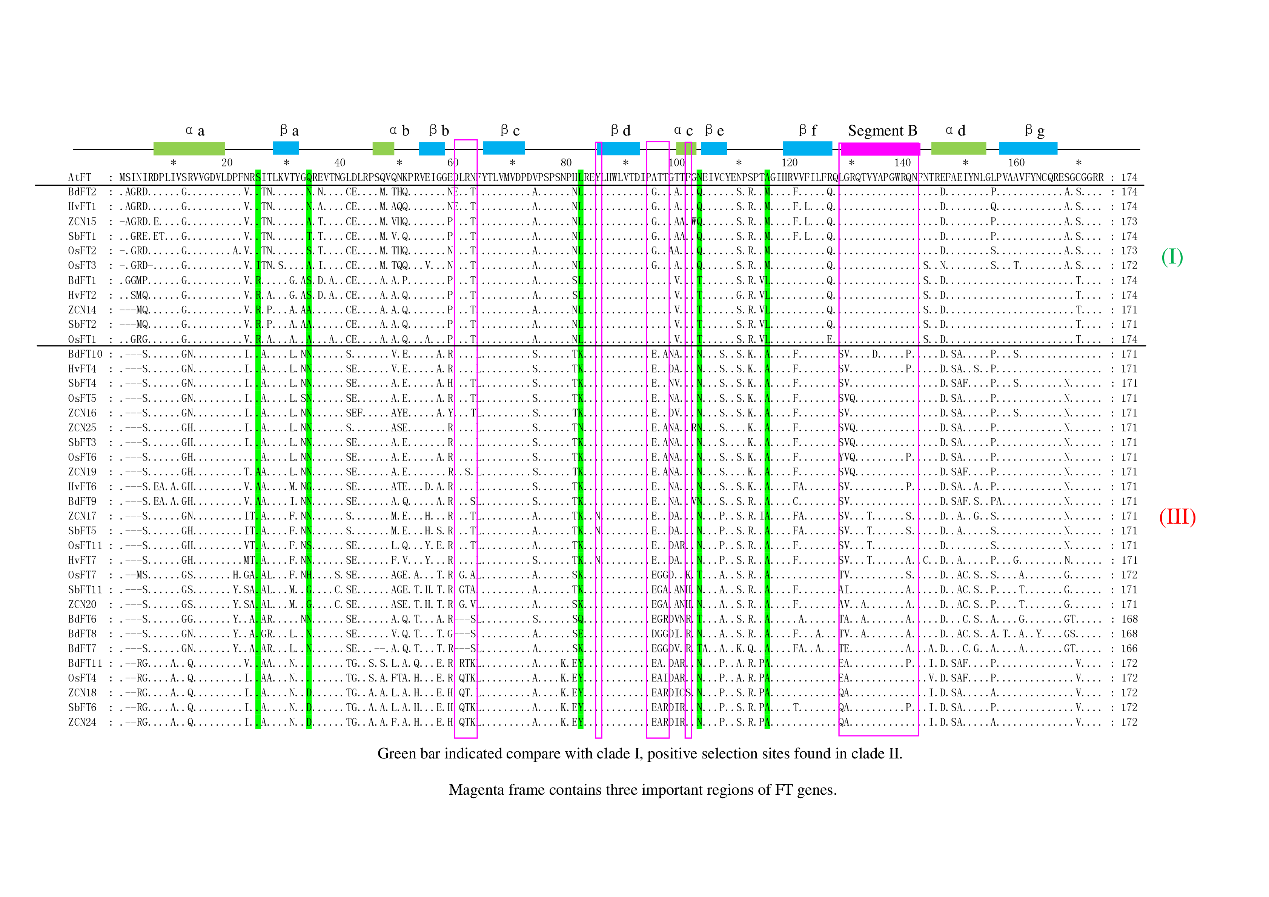
Supplementary Figure 5.** Sequences alignment between I and III clades.

**
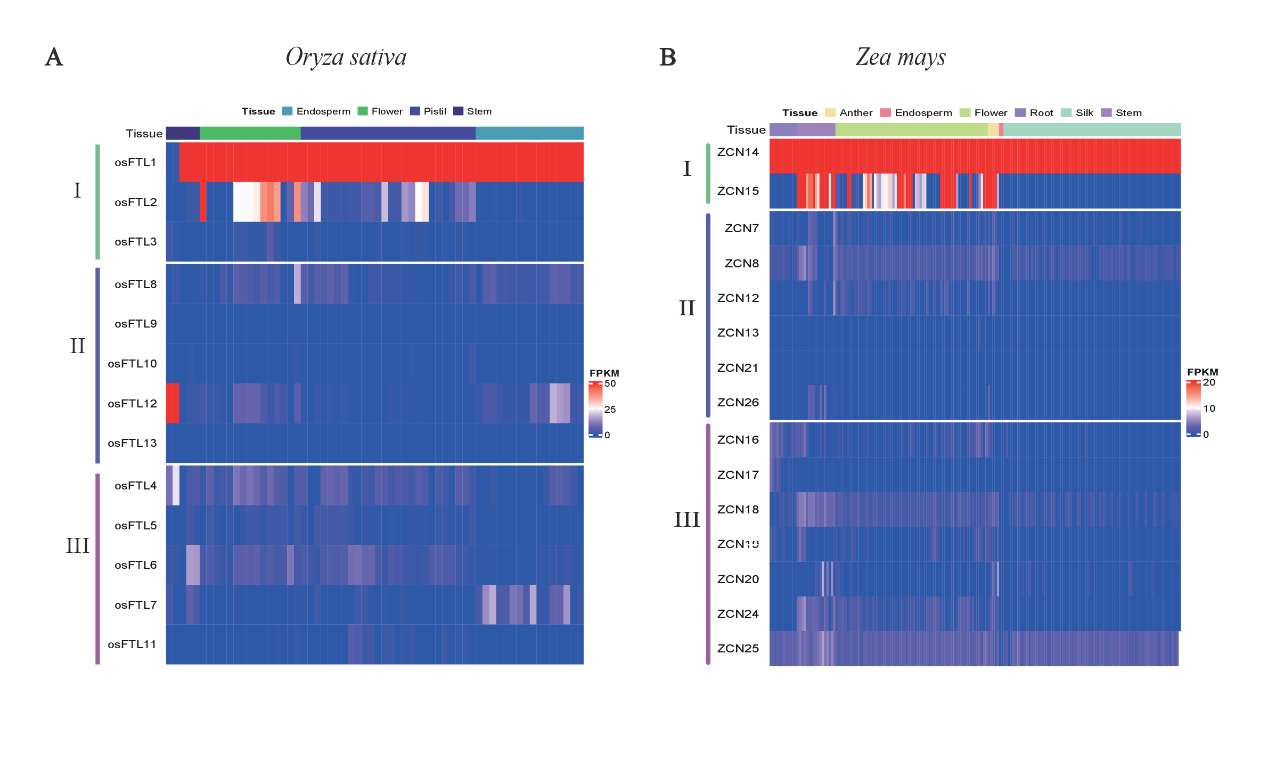
Supplementary Figure 6.** The expression patterns of the three clades FT gene in different monocot species including (A) *Oryza sativa* and (B) *Zea mays*.

**
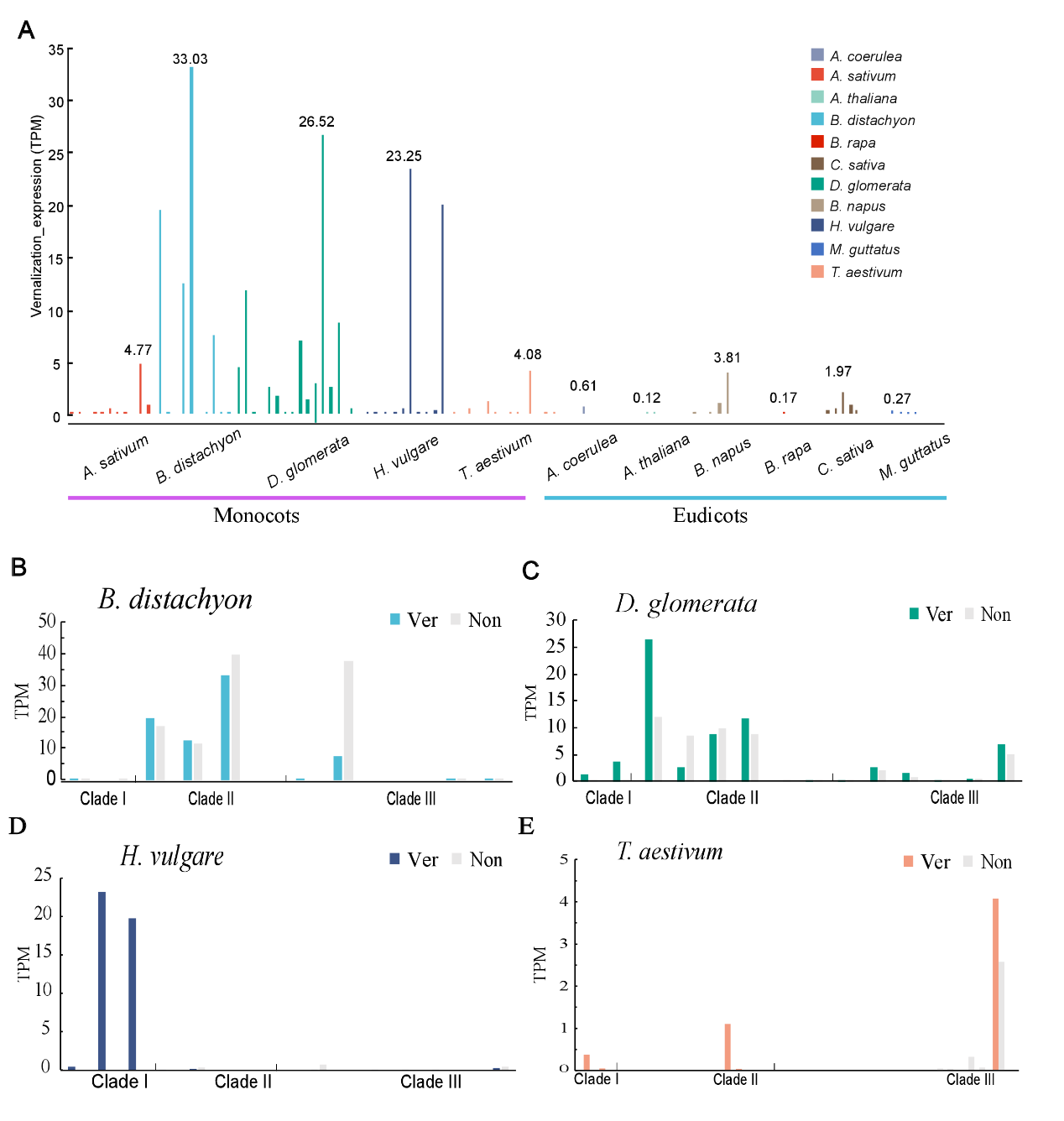
**

**Supplementary Figure 7.** RNA seq analysis of *FT* genes under vernalization.

**
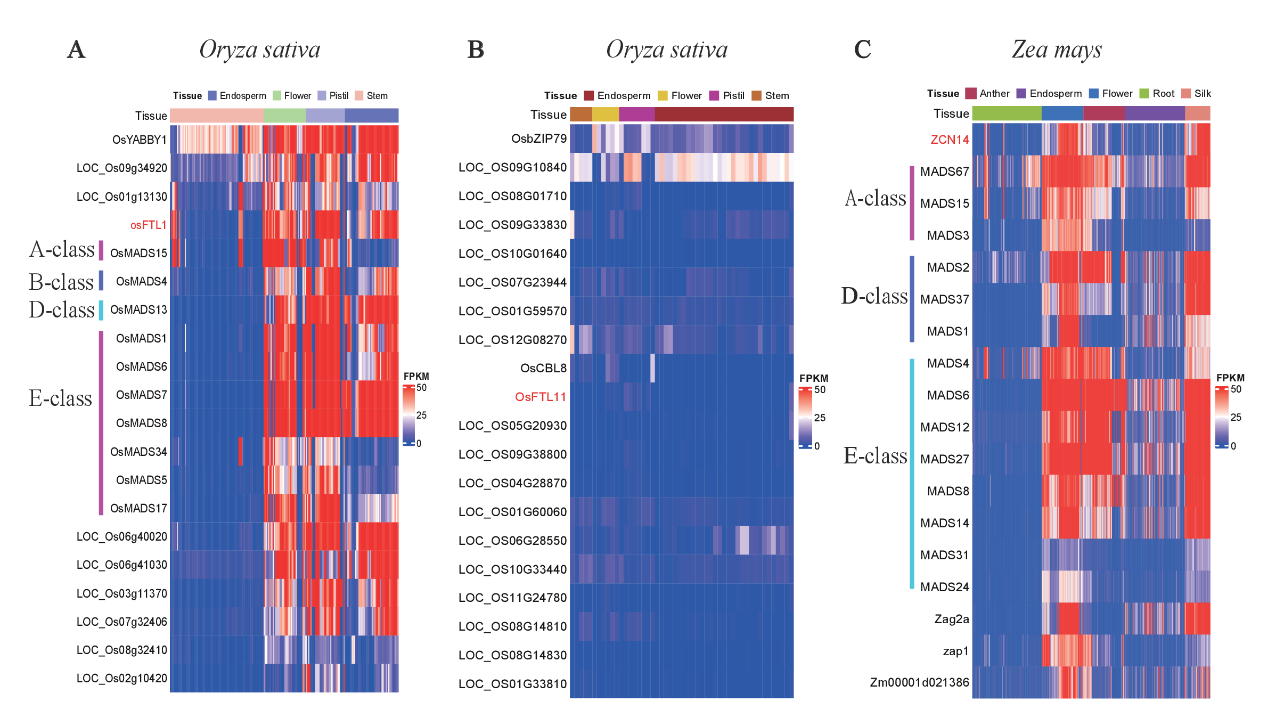
**

**Supplementary Figure 8.** Expression patterns of *FT* and their co-expression genes in *Oryza sativa* and *Zea mays*.

(A) Expression patterns of *osFTL1* and its co-expression genes in endosperm, flower, pistil, and stem, different *MADS-box* class labeled in different color line. (B) Expression patterns of *osFTL11* and its co-expression genes in endosperm, flower, pistil, and stem, different *MADS-box* class labeled in different color line. (C) Expression patterns of *ZCN14* and its co-expression genes in anther, endosperm, flower, root, silk, different *MADS-box* class labeled in different color line.

| **Supplemental Table 1.** Data source used in this study   \| **Species name** \| **Source** \| \| --- \| --- \| \| *Ananas comosus* \| <https://phytozome-next.jgi.doe.gov/info/Acomosus_v3> \| \| *Brachypodium distachyon* \| <https://phytozome-next.jgi.doe.gov/info/Bdistachyon_v3_1> \| \| *Musa acuminata* \| <https://phytozome-next.jgi.doe.gov/info/Macuminata_v1> \| \| *Nymphaea colorata* \| <https://phytozome-next.jgi.doe.gov/info/Ncolorata_v1_2> \| \| *Oryza sativa* \| <https://phytozome-next.jgi.doe.gov/info/Osativa_v7_0> \| \| *Setaria italica* \| <https://phytozome-next.jgi.doe.gov/info/Sitalica_v2_1> \| \| *Sorghum bicolor* \| https://phytozome-next.jgi.doe.gov/info/Sbicolor_v3_1_1 \| \| *Spirodela polyrhiza* \| <https://phytozome-next.jgi.doe.gov/info/Spolyrhiza_v2> \| \| *Triticum aestivum* \| https://phytozome-next.jgi.doe.gov/info/Taestivum_v2_2 \| \| *Zea mays* \| https://phytozome-next.jgi.doe.gov/info/Zmays_RefGen_V4 \| \| *Zostera marina* \| <https://phytozome-next.jgi.doe.gov/info/Zmarina_v2_2> \| \| *Zostera muelleri* \| <https://appliedbioinformatics.com.au/Edwards/index.php/Seagrass_Zmu_Genome> \| \| *Aquilegia coerulea* \| https://phytozome-next.jgi.doe.gov/info/Acoerulea_v3_1 \| \| *Arabidopsis lyrata* \| <https://phytozome-next.jgi.doe.gov/info/Alyrata_v2_1> \| \| *Arabidopsis thaliana* \| <https://phytozome-next.jgi.doe.gov/info/Athaliana_TAIR10> \| \| *Beta vulgaris* \| <https://www.ncbi.nlm.nih.gov/sra?term=SRP023136> \| \| *Brassica oleracea* \| <https://phytozome-next.jgi.doe.gov/info/Boleraceacapitata_v1_0> \| \| *Cajanus cajan* \| <http://www.icrisat.org/gt-bt/iipg/genomedata.zip> \| \| *Capsella rubella* \| <https://phytozome-next.jgi.doe.gov/info/Crubella_v1_0> \| \| *Carica papaya* \| <https://phytozome-next.jgi.doe.gov/info/Cpapaya_ASGPBv0_4> \| \| *Citrus clementina* \| <https://phytozome-next.jgi.doe.gov/info/Cclementina_v1_0> \| \| *Cucumis sativus* \| <https://phytozome-next.jgi.doe.gov/info/Csativus_v1_0> \| \| *Fragaria vesca* \| <https://phytozome-next.jgi.doe.gov/info/Fvesca_v2_0_a2> \| \| *Gossypium raimondii* \| <https://phytozome-next.jgi.doe.gov/info/Graimondii_v2_1> \| \| *Lepidium meyenii* \| [http://www.herbal-genome.cn](http://www.herbal-genome.cn/) \| \| *Linum usitatissimum* \| <https://phytozome-next.jgi.doe.gov/info/Lusitatissimum_v1_0> \| \| *Malus domestica* \| <https://phytozome-next.jgi.doe.gov/info/Mdomestica_v1_1> \| \| *Medicago truncatula* \| <https://phytozome-next.jgi.doe.gov/info/Mtruncatula_Mt4_0v1> \| \| *Nelumbo nucifera* \| <https://www.ncbi.nlm.nih.gov/Traces/wgs/?val=AQOG01> \| \| *Phaseolus vulgaris* \| <https://phytozome-next.jgi.doe.gov/info/Bvulgarisssp_vulgaris_EL10_2_2> \| \| *Populus trichocarpa* \| <https://phytozome-next.jgi.doe.gov/info/Ptrichocarpa_v4_1> \| \| *Prunus mume* \| http:/www.rosaceae.org \| \| *Pyrus bretschneideri* \| http:/www.rosaceae.org \| \| *Solanum lycopersicum* \| <https://phytozome-next.jgi.doe.gov/info/Slycopersicum_ITAG2_4> \| \| *Solanum tuberosum* \| <https://phytozome-next.jgi.doe.gov/info/Stuberosum_v4_03> \| \| *Theobroma cacao* \| <https://phytozome-next.jgi.doe.gov/info/Tcacao_v1_1> \| \| *Vitis vinifera* \| <https://phytozome-next.jgi.doe.gov/info/Vvinifera_v2_1> \|   **Supplemental Table 2.** Gene identifiers used in this study. Genes are obtained from Phytozome database. | | | | |
| --- | --- | --- | --- | --- | --- | --- | --- | --- | --- | --- | --- | --- | --- | --- | --- | --- | --- | --- | --- | --- | --- | --- | --- | --- | --- | --- | --- | --- | --- | --- | --- | --- | --- | --- | --- | --- | --- | --- | --- | --- | --- | --- | --- | --- | --- | --- | --- | --- | --- | --- | --- | --- | --- | --- | --- | --- | --- | --- | --- | --- | --- | --- | --- | --- | --- | --- | --- | --- | --- | --- | --- | --- | --- | --- | --- | --- | --- | --- | --- | --- |
| Species | FT | Gene identifier | TFL1 | MFT |
| Rice | osFTL1 | LOC_Os01g11940 | LOC Os02g32950.1 | LOC Os01g02120.1 |
|  | osFTL2 | LOC_Os06g06320 | LOC Os04g33570.1 | LOC Os06g30370.1 |
|  | osFTL3 | LOC_Os06g06300 | LOC Os11g05470.1 |  |
|  | osFTL4 | LOC_Os09g33850 | LOC Os12g05590.1 |  |
|  | osFTL5 | LOC_Os02g39064 |  |  |
|  | osFTL6 | LOC_Os04g41130 |  |  |
|  | osFTL7 | LOC_Os12g13030 |  |  |
|  | osFTL8 | LOC_Os01g10590 |  |  |
|  | osFTL9 | LOC_Os01g54490 |  |  |
|  | osFTL10 | LOC_Os05g44180 |  |  |
|  | osFTL11 | LOC_Os11g18870 |  |  |
|  | osFTL12 | LOC_Os06g35940 |  |  |
|  | osFTL13 | LOC_Os02g13830 |  |  |
| Maize | ZCN7 | GRMZM2G141756 | GRMZM2G075081 P01 | GRMZM2G021614 P02 |
|  | ZCN8 | GRMZM2G179264 | GRMZM2G092008 P01 | GRMZM2G059358 P01 |
|  | ZCN12 | GRMZM2G103666 | GRMZM2G132880 P01 | GRMZM2G117057 P01 |
|  | ZCN13 | GRMZM2G108016 | GRMZM2G156079 P01 | GRMZM2G152689 P01 |
|  | ZCN14 | GRMZM2G373928 | AC217051.3 FGP006 |  |
|  | ZCN15 | GRMZM2G051338 |  |  |
|  | ZCN16 | GRMZM2G127121 |  |  |
|  | ZCN17 | GRMZM2G075215 |  |  |
|  | ZCN18 | GRMZM2G158809 |  |  |
|  | ZCN19 | GRMZM2G062052 |  |  |
|  | ZCN20 | AC214791 |  |  |
|  | ZCN21 | GRMZM2G019993 |  |  |
|  | ZCN24 | GRMZM2G440005 |  |  |
|  | ZCN25 | GRMZM2G021560 |  |  |
|  | ZCN26 | GRMZM2G400167 |  |  |
| Sorghum | SbFT1 | Sobic.010G045100 | Sobic.004G165100 | Sobic.003G098800 |
|  | SbFT2 | Sobic.003G017200 | Sobic.005G038400 | Sobic.010G136300 |
|  | SbFT3 | Sobic.006G128500 | Sobic.006G068300 |  |
|  | SbFT4 | Sobic.004G206600 | Sobic.008G037300 |  |
|  | SbFT5 | Sobic.K020800 |  |  |
|  | SbFT6 | Sobic.002G262500 |  |  |
|  | SbFT7 | Sobic.004G101800 |  |  |
|  | SbFT8 | Sobic.009G199900 |  |  |
|  | SbFT9 | Sobic.010G164200 |  |  |
|  | SbFT10 | Sobic.003G295300 |  |  |
|  | SbFT11 | Sobic.008G082200 |  |  |
|  | SbFT12 | Sobic.006G047700 |  |  |
|  | SbFT13 | Sobic.003G026600 |  |  |
| *Brachypodium distachyon* | BdFT1 | Bd2g07070 | Bradi3g44860.1 | Bradi1g42510.1 |
|  | BdFT2 | Bd1g48830 | Bradi4g42400.1 | Bradi2g01020.1 |
|  | BdFT3 | Bd2g19670 | Bradi5g09270.1 | Bradi2g27860.1 |
|  | BdFT4 | Bd3g08890 |  |  |
|  | BdFT5 | Bd1g38150 |  |  |
|  | BdFT6 | Bd4g39730 |  |  |
|  | BdFT7 | Bd4g39750 |  |  |
|  | BdFT8 | Bd4g39760 |  |  |
|  | BdFT9 | Bd5g14010 |  |  |
|  | BdFT10 | Bd3g48036 |  |  |
|  | BdFT11 | Bd4g35040 |  |  |
|  | BdFT12 | Bd2g49795 |  |  |
| Barley | HvFT1 | MLOC_68576.1 | MLOC 35818.3 | MLOC 13102.1 |
|  | HvFT2 | Hv.17528 | MLOC 44160.1 |  |
|  | HvFT3 | DQ411319 |  |  |
|  | HvFT4 | MLOC_68576.1 |  |  |
|  | HvFT5 | EF012202 |  |  |
|  | HvFT6 | MLOC_64619.2 |  |  |
|  | HvFT7 | MLOC_58552.3 |  |  |
|  | HvFT8 | MLOC_57326.1 |  |  |
| Pineapple | AcFT1 | Aco010684 | Aco031443.1 | Aco006778.1 |
|  | AcFT2 | Aco010683 |  |  |
|  | AcFT3 | Aco024617 |  |  |
|  | AcFT4 | Aco004292 |  |  |
|  | AcFT5 | Aco006026 |  |  |
|  | AcFT6 | Aco004692 |  |  |
|  | AcFT7 | Aco003470 |  |  |
|  | AcFT8 | Aco008070 |  |  |
| *Spirodela polyrhiza* | SpFT1 | Spipo18G0012000 | Spipo0G0156000 | Spipo8G0058900 |
|  | SpFT2 | Spipo19G0001600 | Spipo9G0034500 |  |
|  | SpFT3 | Spipo14G0029400 |  |  |
|  | SpFT4 | Spipo14G0029500 |  |  |
|  | SpFT5 | Spipo19G0001700 |  |  |
| *Phoenix dactylifera* | PdFT1 | PDK915331 | PDK 30s1140451g003 |  |
|  | PdFT2 | PDK997131 | PDK 30s669531g003 |  |
|  | PdFT3 | PDK715671 | PDK 30s893371g008 |  |
|  | PdFT4 | PDK754971 | PDK 30s893371g009 |  |
|  | PdFT5 | PDK1179671 |  |  |
| *Phalaenopsis equestris* | PeFT1 | PEQU19304 | PEQU 06435 |  |
|  | PeFT2 | PEQU33463 |  |  |
|  | PeFT3 | PEQU33459 |  |  |
|  | PeFT4 | PEQU08401 |  |  |
|  | PeFT5 | PEQU16731 |  |  |
|  | PeFT6 | PEQU06787 |  |  |
|  | PeFT7 | PEQU15502 |  |  |
| *Musa acuminata* | MaFT1 | GSMUA Achr2P19310 | GSMUA Achr10P10290 | GSMUA Achr3P20880 |
|  | MaFT2 | GSMUA Achr10P21780 | GSMUA Achr10P15580 | GSMUA Achr6P09410 |
|  | MaFT3 | GSMUA Achr10P21760 | GSMUA Achr11P21720 | GSMUA AchrUn |
|  | MaFT4 | GSMUA Achr5P06600 | GSMUA Achr3P03070 |  |
|  | MaFT5 | GSMUA Achr5P06420 | GSMUA Achr3P22720 |  |
|  | MaFT6 | GSMUA Achr2P19360 | GSMUA Achr5P20040 |  |
|  | MaFT7 | GSMUA Achr2P20900 | GSMUA Achr8P07730 |  |
|  | MaFT8 | GSMUA Achr3P02730 |  |  |
|  | MaFT9 | GSMUA Achr10P16460 |  |  |
|  | MaFT10 | GSMUA Achr9P07900 |  |  |
|  | MaFT11 | GSMUA Achr2P05870 |  |  |
|  | MaFT12 | GSMUA Achr3P21510 |  |  |
|  | MaFT13 | GSMUA Achr1P17050 |  |  |
| *Picea abies* | PaFT1 | JN039333 |  |  |

| **Supplemental Table 3.** Likelihood ratio test (LRT) of branch model in Ⅱ clade | | | | | | |
| --- | --- | --- | --- | --- | --- | --- |
| Model | LnL | Parameter estimates ω | | | | P-value |
|  |  | Ⅱ -1a | Ⅱ -1b | Ⅱ -2c | Ⅱ -2d |  |
| Model 2 | -10132.3 | 0.3727 | 0.1568 | 0.1636 | 0.1305 | 0.8399 |
| Model 0 | -10133 | 0.1714 |  |  |  |  |

| **Supplemental Table 4.** Likelihood ratio test (LRT) of branch site model for II clade | | | | | | | | | |
| --- | --- | --- | --- | --- | --- | --- | --- | --- | --- |
| Clades | Model | LnL | Parameter estimates | | | | | Positive selection sites | P-value |
| Ⅱ-1a | Model A | -10038.2 | Site class | 0 | 1 | 2a | 2b | 107R 0.609 | 0.0094** |
|  |  |  | f | 0.69259 | 0.28497 | 0.0159 | 0.00654 | 144T 0.938 |  |
|  |  |  | ω0 | 0.11739 | 1 | 0.11739 | 1 |  |  |
|  |  |  | ω1 | 0.11739 | 1 | 999 | 999 |  |  |
|  | Model A null | -10041.6 | 1 |  |  |  |  |  |  |
| Ⅱ -1b | Model A | -10041.8 | Site class | 0 | 1 | 2a | 2b | Not found | 0.9988 |
|  |  |  | f | 0.71449 | 0.28551 | 0 | 0 |  |  |
|  |  |  | ω0 | 0.11927 | 1 | 0.11927 | 1 |  |  |
|  |  |  | ω1 | 0.11927 | 1 | 1 | 1 |  |  |
|  | Model A null | -10041.8 | 1 |  |  |  |  |  |  |
| Ⅱ -2c | Model A | -10041.8 | Site class | 0 | 1 | 2a | 2b | 91T 0.552 | 1 |
|  |  |  | f | 0.65927 | 0.26412 | 0.0547 | 0.02191 |  |  |
|  |  |  | ω0 | 0.11898 | 1 | 0.11898 | 1 |  |  |
|  |  |  | ω1 | 0.11898 | 1 | 1 | 1 |  |  |
|  | Model A null | -10041.8 | 1 |  |  |  |  |  |  |
| Ⅱ -2d | Model A | -10041.8 | Site class | 0 | 1 | 2a | 2b | Not found | 1 |
|  |  |  | f | 0.71449 | 0.28551 | 0 | 0 |  |  |
|  |  |  | ω0 | 0.11927 | 1 | 0.11927 | 1 |  |  |
|  |  |  | ω1 | 0.11927 | 1 | 1 | 1 |  |  |
|  | Model A null | -10041.8 | 1 |  |  |  |  |  |  |

| **Supplemental Table 5.** Likelihood ratio test (LRT) of branch site models for II-1a in II-1 subclade | | | | | | | | | |
| --- | --- | --- | --- | --- | --- | --- | --- | --- | --- |
| Clades | Model | LnL | Parameter estimates | | | | | Positive selection sites | P-value |
| Ⅱ -1a | model A | -4946.51332 | Site class | 0 | 1 | 2a | 2b | 14 V 0.686 |  |
|  |  |  | f | 0.80957 | 0.14641 | 0.03728 | 0.00674 | 144 T 0.739 151 Y 0.551 |  |
|  |  |  | ω0 | 0.09186 | 1 | 0.09186 | 1 | 155 L 0.970* 167 E 0.533 | 0.02412* |
|  |  |  | ω1 | 0.09186 | 1 | 998.983 | 998.983 | 170 C 0.688 |  |
|  | model A null | -4949.05644 | 1 |  |  |  |  |  |  |

| **Supplemental Table 6.** Likelihood ratio test (LRT) of branch model for III-2 clade | | | | | | |
| --- | --- | --- | --- | --- | --- | --- |
| Model | LnL | Parameter estimates ω | | | | P-value |
|  |  | Ⅲ-1a | Ⅲ-1b | Ⅲ-2c | Ⅲ-2d |  |
| Model 2 | -8253.4675 | 0.0001 | 0.1015 | 0.0761 | 0.1514 | 0.05 |
| Model 0 | -8258.2105 | 0.1382 | | | |  |

| **Supplemental Table 7.** Likelihood ratio test (LRT) of branch site model for III-2 clade | | | | | | | | | |
| --- | --- | --- | --- | --- | --- | --- | --- | --- | --- |
| Clades | Model | LnL | Parameter estimates | | | | | Positive selection sites | P-value |
| Ⅲ-2-1a | Model A | -8183.79 | Site class | 0 | 1 | 2a | 2b | Not found | 1 |
|  |  |  | f | 0.89307 | 0.10693 | 0 | 0 |  |  |
|  |  |  | Not found | 1 |  |  |  |  |  |
|  |  |  | ω0 | 0.10747 | 1 | 0.10747 | 1 |  |  |
|  |  |  | ω1 | 0.10747 | 1 | 1 | 1 |  |  |
|  | Model A null | -8183.79 | 1 |  |  |  |  |  |  |
| Ⅲ-2-1b | Model A | -8183.79 | Site class | 0 | 1 | 2a | 2b | Not found | 1 |
|  |  |  | f | 0.89307 | 0.10693 | 0 | 0 |  |  |
|  |  |  | Not found | 1 |  |  |  |  |  |
|  |  |  | ω0 | 0.10747 | 1 | 0.10747 | 1 |  |  |
|  |  |  | ω1 | 0.10747 | 1 | 1 | 1 |  |  |
|  | Model A null | -8183.79 | 1 |  |  |  |  |  |  |
| Ⅲ-2-2c | Model A | -8182.81 | Site class | 0 | 1 | 2a | 2b |  |  |
|  |  |  | f | 0.88237 | 0.10501 | 0.01127 | 0.00134 | 49Q 0.849 55E 0.570 | 0.1998 |
|  |  |  | ω0 | 0.10675 | 1 | 0.10675 | 1 |  |  |
|  |  |  | ω1 | 0.10675 | 1 | 10.5733 | 10.5733 |  |  |
|  | Model A null | -8183.63 | 1 |  |  |  |  |  |  |
| Ⅲ-2-2d | Model A |  | Site class | 0 | 1 | 2a | 2b | 35R 0.895 61L 0.658 96T 0.799 160E 0.542 | 0.1027 |
|  |  | -8181.17 | f | 0.85757 | 0.10297 | 0.03522 | 0.00423 |  |  |
|  |  |  | ω0 | 0.10632 | 1 | 0.10632 | 1 |  |  |
|  |  |  | ω1 | 0.10632 | 1 | 4.28001 | 4.28001 |  |  |
|  | Model A null | -8182.51 | 1 |  |  |  |  |  |  |

| **Supplemental Table 8**. Analysis of function divergence by DIVERGE 3.0 | | | | |
| --- | --- | --- | --- | --- |
| FD | WGD | Subfamilies | Coefficient θ+ SE | No. a |
| Type Ⅰ |  | Ⅰ verse Ⅱ | 0.263430+0.072187 | 7 |
|  |  | Ⅰ verse Ⅲ | 0.420460+0.087306 | 18 |
|  |  | Ⅱ verse Ⅲ | 0.372912+0.076650 | 31 |
|  | τ | Ⅰ -1 verse Ⅰ-2 | 0.923115+0.237917 |  |
|  | τ | Ⅲ-1 verse Ⅲ-2 | 0.175154+0.082966 | 3 |
|  | σ | Ⅱ -1 verse Ⅱ -2 | 0.326298+0.115052 | 3 |
|  | σ | Ⅲ-2-1 verse Ⅲ-2-2 | 0.079862+0.102226 | 2 |
|  | ρ | Ⅱ -1a verse Ⅱ -1b | 0.005497+0.198494 |  |
|  | ρ | Ⅱ -2c verse Ⅱ -2d | 0.258801+0.187799 | 1 |
|  | ρ | Ⅲ-2-1a verse Ⅲ-2-1b | 0.463535+0.156923 | 2 |
|  | ρ | Ⅲ-2-2c verse Ⅲ-2-2d | 0.482516+0.199910 | 8 |
| Type Ⅱ |  | Ⅰ verse Ⅱ | -0.050926 |  |
|  |  | Ⅰ verse Ⅲ | 0.097924 |  |
|  |  | Ⅱ verse Ⅲ | -0.013903 |  |
|  | τ | Ⅰ -1 verse Ⅰ-2 | 0.090365+0.050769 | 13 |
|  | τ | Ⅲ-1 verse Ⅲ-2 | 0.141102 |  |
|  | σ | Ⅱ -1 verse Ⅱ -2 | 0.090495 |  |
|  | σ | Ⅲ-2-1 verse Ⅲ-2-2 | 0.095907 |  |
|  | ρ | Ⅱ -1a verse Ⅱ -1b | 0.001859+0.082501 | 3 |
|  | ρ | Ⅱ -2c verse Ⅱ -2d | 0.057508 |  |
|  | ρ | Ⅲ-2-1a verse Ⅲ-2-1b | 0.039018+0.053039 |  |
|  | ρ | Ⅲ-2-2c verse Ⅲ-2-2d | 0.069112 |  |

| **Supplemental Table 9.** Critical amino acid residues detected with functional divergence in FT gene family | | |
| --- | --- | --- |
|  | Type Ⅰ | Type Ⅱ |
| Ⅰ verse Ⅱ | 13,16,39,53,81,138,149 |  |
| Ⅰ verse Ⅲ | 13,26,28,38,45,53,81,82,97,106,112,115,121,141,144,148,150,152 |  |
| Ⅱ verse Ⅲ | 12,16,18,21,26,38,39,41,49,54,55,74,82,97,103,106,115,123,124,125, 130,131,137,138,146,148,149,150,152,161 |  |
| Ⅰ -1 verse Ⅰ-2 |  | 19,26,27,38,43,49,95,99,103,114,11 5,123,155 |
| Ⅲ-1 verse Ⅲ-2 | 13,25,45 |  |
| Ⅱ -1 verse Ⅱ -2 |  | 16,45,150 |
| Ⅲ-2-1 verse Ⅲ-2-2 | 132,136 |  |
| Ⅱ -1a verse Ⅱ -1b |  | 11,115,155 |
| Ⅱ -2c verse Ⅱ -2d | 115 |  |
| Ⅲ-2-1a verse Ⅲ-2-1b | 107,133 |  |
| Ⅲ-2-2c verse Ⅲ-2-2d | 23,25,30,31,49,98,101,102 |  |
